# Targeted inhibitors of S100A9 alleviate chronic pancreatitis by inhibiting M2 macrophage polarization via the TAOK3-JNK signaling pathway

**DOI:** 10.3389/fimmu.2025.1526813

**Published:** 2025-03-25

**Authors:** Xufeng Tao, Yu Wu, Fangyue Guo, Linlin Lv, Xiaohan Zhai, Dong Shang, Zhan Yu, Hong Xiang, Deshi Dong

**Affiliations:** ^1^ Department of Pharmacy, First Affiliated Hospital of Dalian Medical University, Dalian, China; ^2^ Institute (College) of Integrative Medicine, Dalian Medical University, Dalian, China; ^3^ Laboratory of Integrative Medicine, First Affiliated Hospital of Dalian Medical University, Dalian, China; ^4^ Department of General Surgery, First Affiliated Hospital of Dalian Medical University, Dalian, China

**Keywords:** chronic pancreatitis, M2-type macrophage, S100A9, TAOK3, JNK

## Abstract

**Background:**

Chronic pancreatitis (CP) is a fibro-inflammatory syndrome with unclear pathogenesis and futile therapy. CP’s microenvironment disrupts the fine-tuned balance of macrophage polarization toward a predominance of the M2-like phenotype associated with fibrosis. S100A9 is mainly expressed in monocytes as a potent regulator of macrophage phenotype and function. Here, we investigated the S100A9-related mechanisms underlying CP pathology induced by macrophages polarization.

**Methods:**

*S100a9* knockout (*S100a9*_-/-_) mice and an *in vitro* coculture system of macrophages overexpressing *S100a9* and primary PSCs were constructed to investigate the effects and mechanisms of S100A9-mediated macrophage polarization on pancreatic inflammation and fibrosis underpinning CP pathology. Furthermore, a variety of S100A9-targeted small-molecule compounds were screened from U.S. Food and Drug Administration (FDA)-listed drug libraries through molecular docking and virtual screening techniques.

**Results:**

In CP progression, S100A9 upregulation induces M2 macrophage polarization to accelerate fibrosis via thousand-and-one amino acid kinase 3 (TAOK3)-c-Jun N-terminal kinase (JNK) signaling pathway, and loss of S100A9 reduces CP injury *in vitro* and *in vivo*. Coimmunoprecipitation (co-IP) and molecular docking experiments proved that S100A9 may interact directly with TAOK3 through salt bridges and hydrogen bonding interactions of the residues in the S100A9 protein. Furthermore, cobamamide and daptomycin, as inactivators of the S100A9-TAOK3 interaction, can improve CP by inhibiting the polarization of M2 macrophages.

**Conclusions:**

S100A9 is a significant promoter of M2-like macrophage-induced fibrosis in CP via the TAOK3-JNK signaling pathway. Cobamamide and daptomycin, targeted inhibitors of the S100A9-TAOK3 interaction, may become candidate drugs for CP immunotherapy.

## Introduction

Chronic pancreatitis (CP) is described as a pathologic fibroinflammatory syndrome of the pancreas with progressive morphologic and functional changes, resulting in chronic pain, pancreatic insufficiency, and elevated risk of pancreatic ductal adenocarcinoma (1, 2). Pancreatic fibrosis is a major pathological feature of typical CP, which is manifested by the abnormal deposition of extracellular matrix (ECM) proteins secreted by activated pancreatic stellate cells (PSCs) (3, 4). According to the advanced “two-hit” hypothesis, CP mostly begins with recurrent bouts of acute inflammation of the pancreas parenchyma, and cells of the innate immune system infiltrate the damaged pancreas. In particular, macrophages represent a population of cells within the innate immune system with vast plasticity and can therefore activate PSCs, and ongoing injury or stress drives fibrosis through activated immune cells. Macrophages, especially its selectively activated subtype (M2), play a key role in the regulation of PSCs-mediated fibrosis (5, 6). In CP, M2-type macrophages activate PSCs by releasing transforming growth factor β(TGF-β) and platelet-derived growth factor (PDGF), which leads to the imbalance of extracellular matrix (ECM) caused by excessive deposition (7–9). Although the crosstalk between macrophages and PSCs has been revealed, it is still largely undefined (10).

S100 calcium binding protein A9 (S100A9), also referred to as myeloid-related protein 14 (MRP-14), is mainly expressed in neutrophils and monocytes as one of the most abundant damage-associated molecular patterns (DAMPs) (11). S100A9 serves as a potential biomarker for diagnosis and an early warning indicator of therapeutic responses to diseases associated with inflammation (12). Our previous research also reported that knockout of S100a9 in mice significantly reduced acute pancreatitis (AP)-induced inflammation and structural damage to the pancreatic parenchyma (13). And S100A9 can exert a variety of immune functions by inducing monocyte/macrophage migration and activation (14, 15). It is found that S100A9 can dynamically regulate the polarization direction of macrophages to pro-inflammatory M1-type (such as stimulated by LPS/IFN-γ) or pro-repair M2-type (such as IL-4/IL-13 dominant environment) through microenvironment signals (16–18). Moreover, tasquinimod (a small molecule inhibiting S100A9 signaling) can induce a polarization switch from M2-like to M1-like macrophages (19). Although S100A9 is a well-known regulator of macrophage phenotype and function, to date, its regulatory effect is controversial (20, 21). Little is known about S100A9-mediated macrophage polarization in CP progression.

In this study, *S100a9* knockout mice (*S100a9* ^-/-^ C57BL/6 mice) and an *in vitro* coculture system with macrophages overexpressing *S100a9* and primary PSCs were constructed to clarify whether and how S100A9 regulates the M2-type polarization of macrophages that contributes to pancreatic inflammation and fibrosis in CP progression. Furthermore, based on molecular docking and virtual screening technologies, U.S. Food and Drug Administration (FDA)-listed drug libraries were used as a source for screening S100A9-targeted drugs for CP therapy. New molecular targets and candidate drugs for the precise treatment of CP will provide increased hope to patients.

## Materials and methods

### Animals

Adult male wild-type (WT, n=10) and *S100a9* knockout (*S100a9* ^-/-^, n=10) C57BL/6 mice (6-8 weeks, body weight 18-22 g) were provided by Cyagen Biosciences (Guangzhou, China). All mice were maintained under a 12-h light-dark cycle in a temperature-controlled (25 ± 2°C) room. The model of CP was established by intraperitoneal injection of cerulein as previously described (22). In brief, mice were intraperitoneally injected with 50 Mg/kg cerulein (Meilunbio; Dalian, China) every Monday, Wednesday and Friday, 6 times/day with an interval of 1 h, for 8 weeks.

The steps for constructing *S100a9* knockout mice were as follows: the *S100a9* gene (NCBI Reference Sequence: NM_009114.3) is located on mouse chromosome 3. Using CRISPR/Cas9 technology, sgRNA was designed. Then, sgRNA and Cas9 mRNA were injected into the fertilized eggs of C57BL/6 mice through high-throughput electroporation. After embryo transfer, mouse *S100a9* gene knockout was detected using PCR and sequencing methods (**Supplementary Figures S1A, B**).

### Enzyme-linked immunosorbent assay

S100A9 in mouse serum was detected using an S100A9 enzyme linked immunosorbent assay (ELISA) kit (R&D Systems, MN, USA).

### Cell culture

The human embryonic kidney cell line 293T and RAW264.7 cells were purchased from the American Type Culture Collection (ATCC, Manassas, VA, USA) and were cultured in Dulbecco’s Modified Eagle Medium (DMEM) containing 10% fetal bovine serum. Mouse bone marrow-derived macrophages (BMDMs) were obtained from C57BL/6 mice and also cultured in DMEM containing 10% fetal bovine serum (23).

### Flow cytometry

Pancreatic tissue cells, BMDMs, or mouse mononuclear macrophage cell line RAW264.7 were fixed and permeabilized with Fixation and Permeabilization Solution (BD Biosciences; Franklin Lakes, New Jersey, USA). Then, anti-F4-80 (1:100; Santa Cruz Biotechnology; TX, USA), anti-CD68 (1:100; Santa Cruz Biotechnology; TX, USA), anti-S100A9 (1:100; Cell Signaling Technologies; MA, USA) or anti-CD206 (1:100; Santa Cruz Biotechnology; TX, USA) was added for staining. Finally, the contents were detected by flow cytometry.

### Histology, immunohistochemistry, and immunofluorescence

Mouse pancreas fixed in 4% paraformaldehyde was embedded in paraffin, sectioned, and placed on glass slides. Hematoxylin and eosin (H&E) or the Servicebio^®^ Masson staining kit (Servicebio; Wuhan, China) was used for staining. In addition, slides were incubated with corresponding primary antibodies overnight at 4°C for immunofluorescence labeling (anti-F4-80, anti-CD68, and anti-CD206 1:100; Santa Cruz Biotechnology; TX, USA, anti-S100A9 1:100; Cell Signaling Technologies; MA, USA or anti-α-SMA 1:200; Wanleibio; Shenyang, China) and at 37°C for 1 h with fluorescent secondary antibodies. Finally, the sample images were visualized, and the effects of CP on pancreatic damage and fibrosis were evaluated according to the scoring system (**Supplementary Table S1**).

### Plasmid transfection experiment

The transfection plasmid containing shRNA-S100A9 (*shS100a9*) or shRNA-TAOK3 (*shTaok3*) (GenePharma; Shanghai, China) was used for gene silencing, and the plasmid containing S100A9-DNA (*S100a9*), TAOK3-DNA (*Taok3*) (Hanbio; Shanghai, China) or TAOK3-DNA-MU (*Taok3*-MU) (GenePharma; Shanghai, China) was used for gene overexpression. Cells transfected with empty plasmid were used as negative control (NC).

### Cell adhesion and migration assay

In the adhesion assay, we used Matrigel matrix (Corning, USA) that was preplated in 96-well plates in a 37°C incubator for 1 h. RAW264.7 cells were suspended in serum-free medium and seeded in 96-well plates preplated with Matrigel matrix for incubation at 37°C for 1 h. Next, the cells were incubated with 50 Ml 3-(4,5-dimethyl-2-thiazolyl)-2,5-diphenyltetrazolium-bromide (MTT) (Solarbio; Beijing, China) for 4 h. Then MTT was removed and 200 Ml dimethyl sulfoxide (DMSO) (Solarbio; Beijing, China) was added to each well. Finally, the optical density (OD) value of each well was measured using an enzyme-labeled instrument (BioTek; VT, USA) at 570 nm, and statistical analysis was performed according to the ratio of OD570 _treated group_/OD570 _untreated group_.

In addition, in the migration assay, we used a pipette to vertically scratch a 6-well plate filled with RAW264.7 cells. Then, images were collected at 0 h and 48 h. Finally, the scratch area was calculated by ImageJ software (NIH; Bethesda, USA), and statistical analysis was performed according to the ratio of (initial area-area at a certain time point)/initial area.

### Quantitative real-time PCR analysis

RNAex Pro RNA reagent (Accurate Biology; Hunan, China) was used to isolate and extract total cell RNA; then, an Evo M-MLV RT MIX Kit (Accurate Biology; Hunan, China) was used to synthesize cDNA. Finally, the SYBR^®^ Green Premix Pro Taq HS qPCR Kit (Accurate Biology; Hunan, China) was used to quantify the mRNA expression levels of different genes in the ABI 7500 real-time PCR system (Applied Biosystems; CA, USA). The primer sequences are shown in **Supplementary Table S2**.

### Coculture of macrophages with primary PSCs

The mouse pancreas was cut into tissue pieces of approximately 1 mm^3^ in size, spread evenly on the bottom of the culture plate, and then placed in a 5% CO_2_, 37°C incubator for 30 min to attach the tissue pieces against the wall. Finally, DMEM culture medium containing 20% fetal bovine serum and 0.01% soybean trypsin inhibitor was added, and the culture medium was changed daily. After 3 days, the primary PSCs were cocultured with the supernatant of RAW264.7 cells overexpressing *S100a9* for 24 h and then were stained with α-SMA antibody (1:200; Wanleibio; Shenyang, China) by immunofluorescence. Finally, the staining of PSCs was observed under an Olympus IX73 fluorescence microscope (Olympus; Tokyo, Japan).

### Transcriptomic analysis

Using the polyA structure at the 3′-terminal end of messenger RNA and related molecular biology techniques, the complete total RNA of RAW264.7 cells or 293T cells was subjected to mRNA isolation, fragmentation, double-stranded cDNA synthesis, cDNA fragmentation modification, magnetic bead purification, fragmentation sorting and library expansion. After quality control, a sequencing library suitable for the Illumina platform was finally obtained. RNA-seq data were analyzed for gene structure, expression level, expression difference, gene enrichment, etc. EBseq2 was used for the difference analysis between the two groups, and the screening criteria for the differentially expressed genes were as follows: fold change ≥ 2 and FDR<0.01. Then, the gene ontology (GO) functions and Kyoto Encyclopedia of Genes and Genomes (KEGG) pathways of the differentially expressed genes were analyzed. Finally, the differentially expressed genes are presented in the form of a volcano map, MA map, Wayne map, cluster heatmap, and protein interaction map.

### IP and LC−MS/MS analysis

RAW264.7 cells overexpressing *S100a9* were harvested and lysed. Anti-Flag (1:2000; Applygen; Beijing, China) or IgG antibodies (Abcam; Cambridge, UK) were added to the lysis solution for antibody immobilization at 4°C overnight. After incubation with Protein A/G Magnetic Beads (MedChemExpress; Shanghai, China) at 4°C for 3 h, the protein complex was centrifuged and then washed 3 times with Pierce IP Lysis Buffer (Thermo; MA, USA) for SDS−PAGE analysis. Finally, the SDS−PAGE gel was subjected to silver staining to detect the difference in protein binding between the S100A9 and IgG antibodies. In addition, after pull-down experiments, the two protein samples underwent reductive alkylation and enzymolysis. Moreover, to detect the polypeptide sequence of protein samples, liquid chromatography-tandem mass spectrometry (LC-MS/MS) analysis was implemented. The polypeptide sequence was identified using ProteinPilot software of the AB SCIEX Triple TOF™ 5600 plus MS system (MA, USA).

### Co-IP

RAW264.7 cells overexpressing *S100a9* and 293T cells overexpressing *Taok3* were lysed with cell lysis buffer (Solarbio; Beijing, China), and the supernatant was collected. Then, S100A9 (1:1000; proteintech; Wuhan, China), SLK (1:500; Santa Cruz Biotechnology; TX, USA), TAOK3 (1:3000; Thermo Fisher Scientific; MA, USA), Flag (1:2000; Applygen; Beijing, China), HA (1:2000; Applygen; Beijing, China) or IgG (Abcam; Cambridge, UK) antibodies were added to the supernatant and incubated at 4°C overnight. After that, the supernatant was incubated with Protein A/G Magnetic Beads (MedChemExpress; Shanghai, China) at 4 °C for 4 h. Subsequently, the magnetic beads were separated, and the supernatant was collected. Finally, Pierce IP Lysis Buffer (Thermo; MA, USA) was added, samples were heated at 100°C for 7 min, and then they were analyzed by western blotting.

### Pull-down

The interaction between the S100A9 protein and TAOK3 protein was directly detected *in vitro* by purified 3×flag-S100A9 protein and HA-TAOK3 protein and then analyzed by western blotting.

### Western blotting

Total protein from RAW264.7 cells was extracted, separated by SDS−PAGE and transferred to PVDF membranes (Millipore; MA, USA). After being sealed with 5% BSA blocking buffer, the membranes were incubated with the primary antibodies S100A9 (1:1000; Cell Signaling Technologies; MA, USA), TAOK3 (1:3000; Thermo Fisher Scientific; MA, USA), JNK (1:1000; Cell Signaling Technologies; MA, USA), p-JNK (1:1000; Cell Signaling Technologies; MA, USA), and β-actin (1:5000; Proteintech; Wuhan, China) overnight at 4°C and then were incubated at room temperature for 1 h with secondary antibody (1:5000; Abbkine; Wuhan, China). Finally, the protein bands were visualized using an imaging system (Tanon 4200; Shanghai, China).

### Molecular docking

Protein−protein docking in ClusPro was used for molecular docking simulation and predicting the binding affinity of TAOK3 for S100A9 protein. For protein docking, the smaller protein (a smaller number of residues) was usually set as the ligand and the other as the receptor. The ligand was rotated by 70,000 rotations. For each rotation, the ligand was translated in the x, y, and z axes relative to the receptor on a grid. One translation with the best score was chosen from each rotation. Of the 70,000 rotations, 1000 rotations/translation combination that had the lowest scores were chosen. Then, greedy clustering of these 1000 ligand positions with a 9 Å C-alpha root mean square deviation (RMSD) radius was performed to find the ligand positions with the most “neighbors” in 9 Å, i.e., cluster centers. The top 10 cluster centers with the most cluster members were then retrieved and inspected visually one by one. The intermolecular contacts from the most likely poses were further evaluated. The docked structures and interface residues were analyzed using MOE v2018.01.

### Virtual screening

Virtual screening was conducted in MOE. The 2D structures of ligands were converted to 3D structures in MOE through energy minimization. The monomer model of TAOK3 was used as the receptor, and the drug compound library (2800 drugs) was used as the VS library. Prior to docking, the force field of AMBER10: EHT and the implicit solvation model of the reaction field (R-field) were selected. The residues involved in the interactions with S100A9 were selected as the binding site. The docking workflow followed the “induced fit” protocol, in which the side chains of the receptor pocket were allowed to move according to ligand conformations, with a constraint on their positions. The weight used for tethering side chain atoms to their original positions was 10. For each ligand, all docked poses were ranked by London dG scoring first, then a force field refinement was carried out on the top 30 poses followed by a rescoring of GBVI/WSA dG. The conformation with the lowest binding free energy was finally identified as the best probable binding mode. Molecular graphics were generated by PyMOL (www.pymol.org).

### Toxicology and pharmacodynamics of S100A9-targeted inhibitors

First, RAW264.7 cells were used to screen the toxicology and pharmacodynamics of small molecular inhibitors through the CCK-8 and flow cytometry assays *in vitro*. Then, a CP mouse model was established according to the above method. To evaluate the toxicology and pharmacodynamics *in vivo*, C57BL/6 mice were randomly divided into 6 groups: Ctrl, CP, cobamamide, daptomycin, CP + cobamamide and CP + daptomycin. After the fourth week of CP modeling, cobamamide and daptomycin were injected intraperitoneally for 4 weeks (cobamamide 0.2 mg/kg/day and daptomycin 50 mg/kg/day). After 8 weeks, serum was collected for liver and kidney toxicity examination, and the heart, liver, spleen, lung, kidney, brain, intestine and pancreas of mice in each group were collected for pathological observation.

### Statistical analysis

Data are expressed as the mean ± standard error of the mean (SEM). All analyses were performed using GraphPad Prism 6.0 software (GraphPad; CA, USA). Differences among multiple groups were determined using one-way analysis of variance (one-way ANOVA), while the differences between two groups were analyzed using an unpaired Student’s *t* test. *P* values of < 0.05 or < 0.01 were considered statistically significant.

## Results

### S100A9 expression level is significantly increased in macrophages in CP

**Figure 1:**
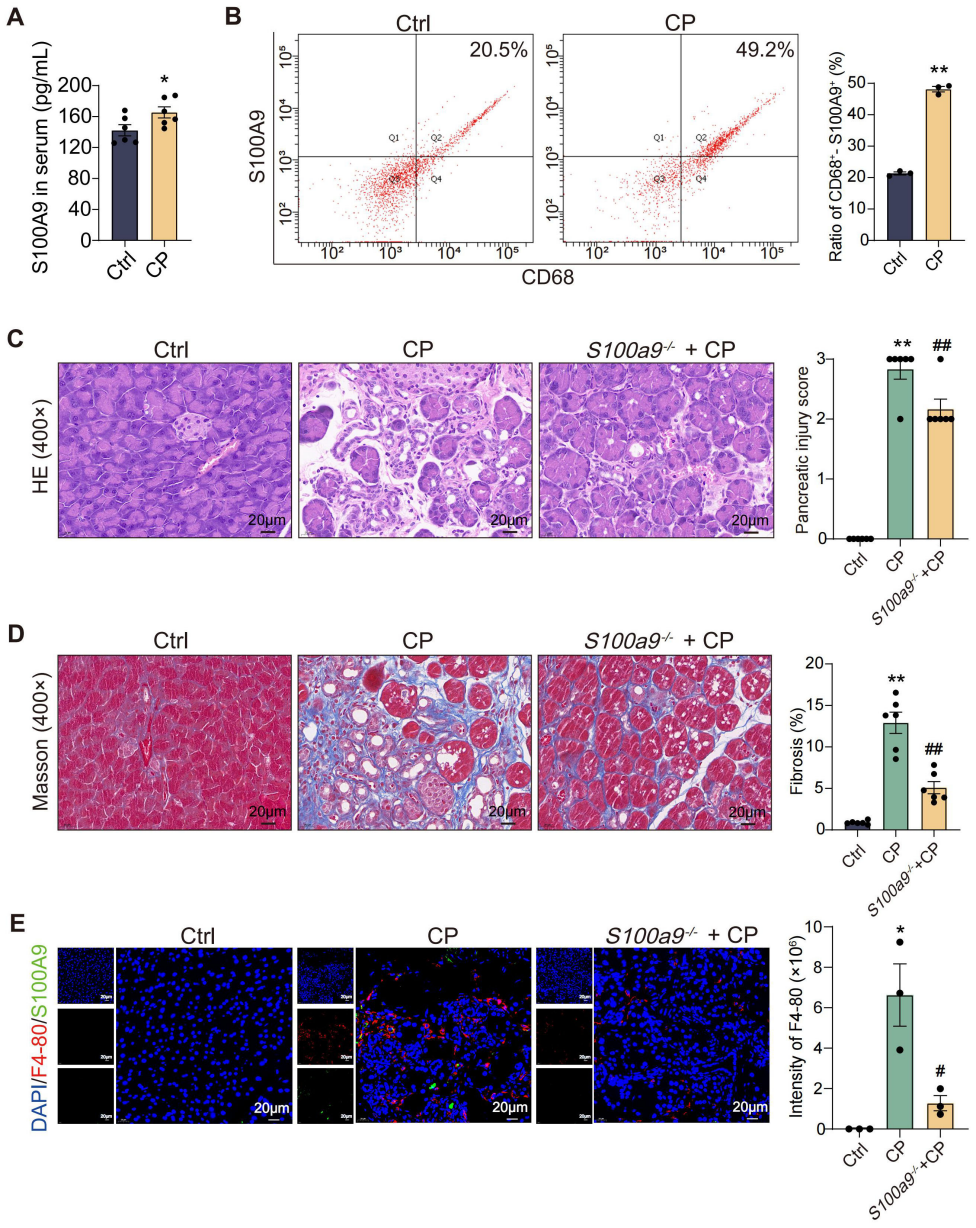
S100A9 expression level is significantly increased in macrophages in CP. **(A)** ELISA results showed that the level of S100A9 in serum of CP mice was high (n=6). **(B)** Flow cytometry showed that the content of S100A9 in macrophages in the pancreas of CP mice was high (n=3). **(C)** HE staining showed that *S100a9^-/-^* had less pancreatic injury (n=6). **(D)** Masson staining showed that the degree of pancreatic fibrosis in *S100a9^-/-^* was lighter (n=6). **(E)** Immunofluorescence staining of F4-80^+^-S100A9^+^ indicated that the expression of S100A9 in infiltrated macrophages was up-regulated. (n=3). Data were presented as the mean ± SEM; *P < 0.05, **P < 0.01 vs. Ctrl mice; ^#^ *P* < 0.05, ^##^ *P* < 0.01 vs. CP mice.

As shown in **Figure 1A**, compared with the Ctrl, the content of S100A9 in the serum of CP mice increased significantly. Additionally, the content of S100A9 in macrophages infiltrating the pancreas of CP mice was higher than that in the Ctrl mice (**Figure 1B**). To further explore the importance of S100A9 in the course of CP, *S100a9* ^-/-^ mice were constructed. H&E staining showed that the pancreatic tissue damage of *S100a9^-/-^* mice was significantly reduced compared to CP mice (**Figure 1C**). Masson staining showed that the positive areas of blue collagen fibers in the pancreatic insults of CP mice were significantly increased, but the *S100a9* ^-/-^ pancreas showed less fibrosis than the CP pancreas (**Figure 1D**). Moreover, the F4-80-positive area (red fluorescent region) was increased in the pancreatic lesions of CP mice compared to the Ctrl, but decreased in the pancreas of *S100a9* ^-/-^ mice compared with CP mice, suggesting that *S100a9* ^-/-^ reduced CP-induced macrophage infiltration (**Figure 1E**). We also found that S100A9-positive area (green fluorescence) of macrophages with pancreatic lesions in CP mice was increased compared with the Ctrl group, indicating that S100A9 expression was up-regulated in macrophages after infiltration (**Figure 1E**). These results suggested that macrophages may accelerate pancreatic injury and fibrosis in CP progression in an S100A9-dependent manner.

### S100A9 promotes adhesion and migration of macrophages *in vitro*

**Figure 2:**
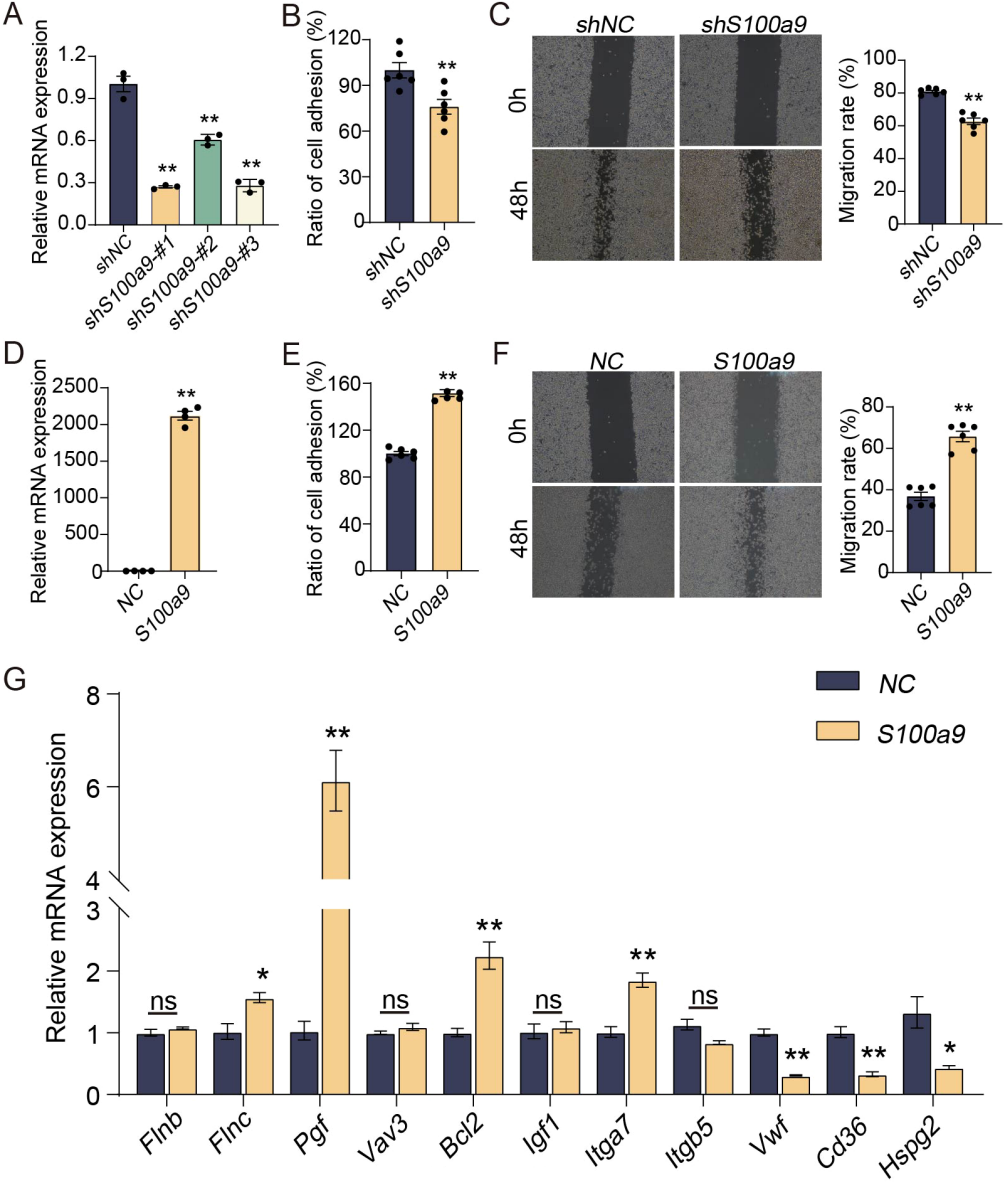
S100A9 promotes adhesion and migration of macrophages *in vitro.* **(A)** sh*S100a9*-#1 significantly downregulated the expression of *S100a9* mRNA in RAW264.7 cells (n=3). **(B)** The adhesion experiment showed that sh*S100a9*-#1 inhibites the adhesion of RAW264.7 cells (n=6). **(C)** The scratch experiment indicated that sh*S100a9*-#1 inhibites the migration of RAW264.7 cells (n=6). **(D)** The expression of *S100a9* mRNA in RAW264.7 cells was significantly upregulated by *S100a9*-DNA (n=4). **(E)** The adhesion experiment showed that the adhesion of RAW264.7 cells overexpressed with *S100a9* is enhanced (n=6). **(F)** The scratch experiment indicated that the migration of RAW264.7 cells overexpressed with *S100a9* is enhanced (n=6) **(G)** qPCR indicated that *S100a9* can promote the expression of adhesion related genes in RAW264.7 cells (n=3 or 4). Data were presented as the mean ± SEM; *P < 0.05, **P < 0.01 vs. NC group.

To observe the effect of S100A9 on macrophages, RAW264.7 cells were selected for study *in vitro*. As shown in **Figure 2A**, the mRNA expression of *S100a9* in RAW264.7 cells was significantly downregulated by *shS100a9*-#1, -#2 and -#3, and the shRNA template sequence of mouse *S100a9* is shown in **Supplementary Table S3**. Considering that *shS100a9*-#1 had the best knockdown efficiency, it was chosen for subsequent macrophage adhesion and migration assays. Compared with those of the NC, the adhesion and migration of RAW264.7 cells with low *S100a9* mRNA expression were significantly decreased (**Figures 2B, C**). In addition, compared with the NC, the adhesion and migration of RAW264.7 cells with high *S100a9* mRNA expression were significantly increased (**Figures 2D–F**). Furthermore, *S100a9* overexpression significantly upregulated the expression of a series of adhesion- and migration-related genes, including *Pdgfrb, Vegfc, Flnc, Pgf, Bcl2*, and *Itga7*, but also significantly downregulated some genes, including *Vwf, Cd36*, and *Hspg2* (**Figure 2G**). Therefore, we can conclude that S100A9 can promote the adhesion and migration of macrophages *in vitro*.

### S100A9 induces M2 polarization of macrophage to promote PSCs activation *in vitro*

**Figure 3:**
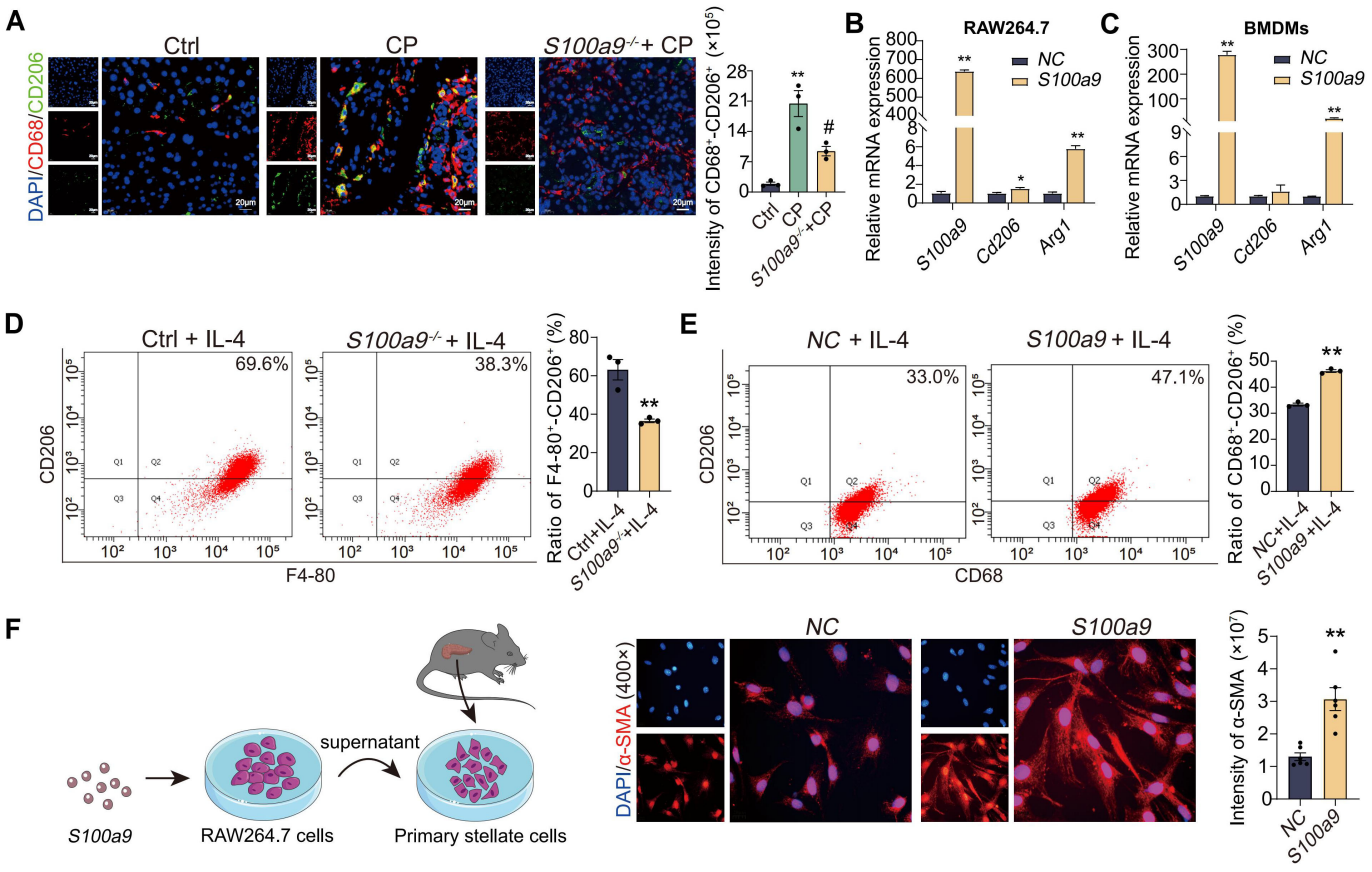
S100A9 induces M2 polarization of macrophage to promote PSCs activation *in vitro.* **(A)** The section of CD68^+^-CD206^+^ fluorescence double staining showed that both CD68 and CD206 protein levels were downregulated in the pancreas of *S100a9* ^-/-^ (n=3). **(B)** qPCR showed that the overexpression of *S100a9* in the RAW264.7 cells increased the mRNA expression of *Arg1* and *Cd206* (n=3). **(C)** qPCR showed that the overexpression of *S100a9* in the BMDMs increased the mRNA expression of *Arg1* and *Cd206* (n=3). **(D)** Flow cytometry showed that the BMDMs from *S100a9^-/-^* mice combined with IL-4 stimulation could reduce the number of M2 macrophages (n=3). **(E)** Flow cytometry showed that overexpression of *S100a9* in the RAW264.7 cells combined with IL-4 stimulation could increase the number of M2 macrophages (n=3). **(F)** α-SMA immunofluorescence showed that the supernatant of RAW264.7 cells overexpressing *S100a9* can induce activation of primary PSCs (n=6). Data were presented as the mean ± SEM; *P < 0.05, **P < 0.01 vs. Ctrl mice or NC group; ^#^ *P* < 0.05 vs. CP mice.

Considering S100A9 has the ability to promote adhesion and migration of macrophages, we speculated that S100A9 could promote the polarization of macrophages. As shown in **Figure 3A**, fluorescence double staining of CD68^+^-CD206^+^ in tissue sections showed that the expression of both CD68 (red fluorescence) and CD206 (green fluorescence) proteins was upregulated in CP mice but downregulated in *S100a9* ^-/-^ mice. These results indicated that *S100a9^-/-^* reduced CP-induced M2 macrophage infiltration. Consistently, the mRNA levels of *Arg1* and *Cd206*, markers of M2 macrophages, were increased in RAW264.7 cells and BMDMs overexpressing *S100a9 in vitro* (**Figures 3B, C**). Flow cytometry showed that S100A9 could increase the percentage of M2-type macrophages after IL-4 (20ng/mL) stimulation (**Figures 3D, E**). To observe the damage caused by macrophages overexpressing *S100a9* to PSCs, an *in vitro* coculture system of RAW264.7 cells overexpressing *S100a9* with primary PSCs was constructed. As shown in **Figure 3F**, in the coculture system of primary PSCs and RAW264.7 cells overexpressing *S100a9*, immunofluorescence showed that α-smooth muscle actin (α-SMA), a marker of PSC activation, was significantly increased in primary PSCs. These results confirm that S100A9 may promote CP progression by promoting M2-type polarization of macrophages.

### S100A9 regulates MAPK signaling pathway through interacting with TAOK3

**Figure 4:**
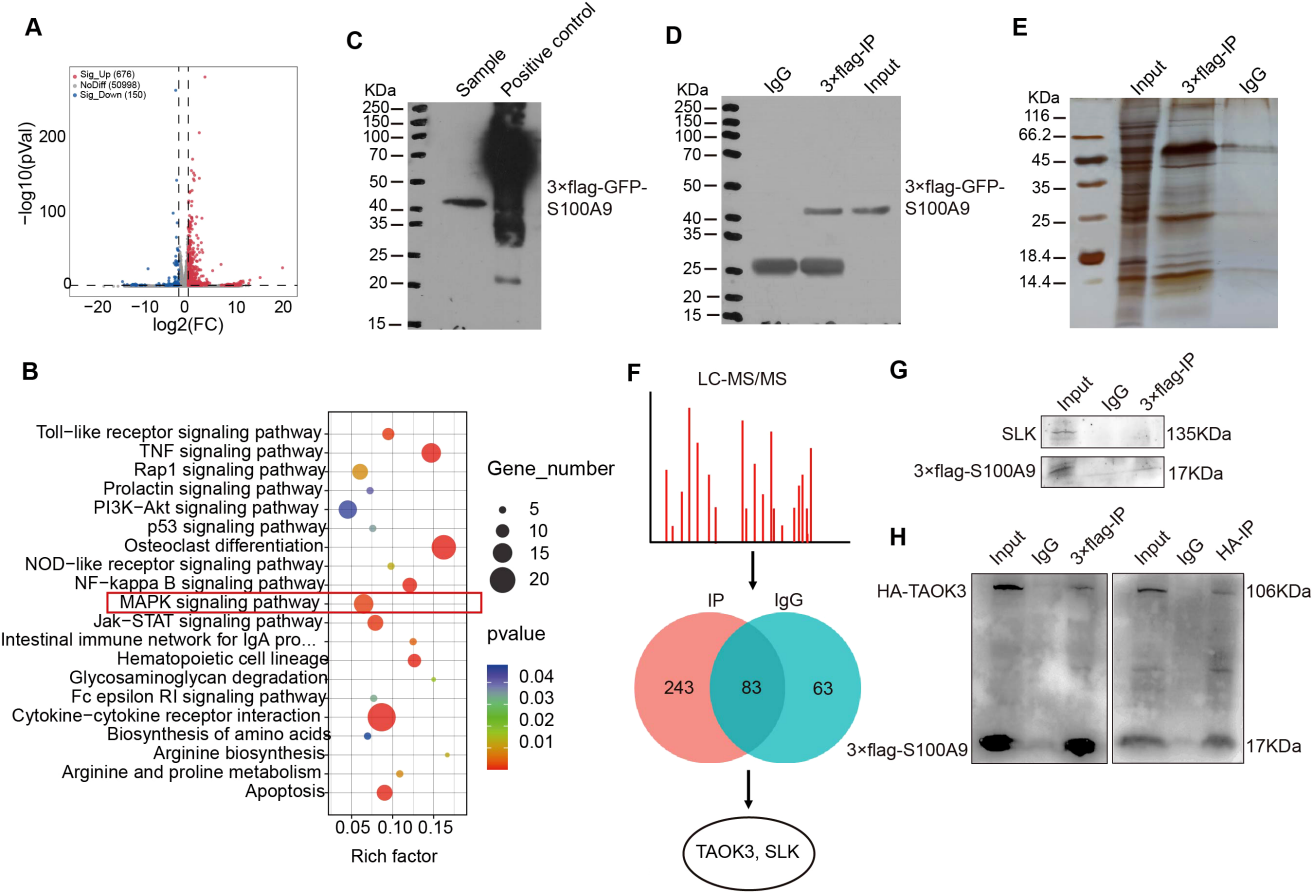
S100A9 regulates MAPK signaling pathway through interacting with TAOK3. **(A)** The volcanic map showed the differentially expressed genes of RNA-seq in RAW264.7 cells overexpressing *S100a9*. **(B)** KEGG analysis showed that *S100a9* regulates MAPK signaling pathway. **(C)** The expression of target protein (S100A9) was detected near 40KDa by western blotting before IP. **(D)** The target protein (S100A9) was detected near 40KDa by western blotting after IP. **(E)** The signal of the target protein was detected near 40KDa using silver staining. **(F)** LC-MS/MS screened and identified proteins interacting with S100A9. **(G)** Co-IP showed that S100A9 protein had no obvious interaction SLK. **(H)** Co-IP showed that S100A9 protein interacted with TAOK3 protein.

To explore the mechanism of S100A9-regulated macrophage polarization, RNA-seq analysis was performed. As shown in **Figure 4A**, there were a total of 826 differentially expressed genes between RAW264.7 cells overexpressing *S100a9* and normal RAW264.7 cells. Among these differentially expressed genes, 676 genes were upregulated, and 150 were downregulated. The top 20 signaling pathways enriched by KEGG are shown in **Figure 4B**. In order to confirm that S100A9 regulates the signaling pathway of M2 macrophages and the downstream molecules of the pathway, we conducted an immunoprecipitation (IP) experiment with S100A9. Before the IP experiment, we tested the expression of the target protein through western blotting, which confirmed the high quality of the samples (**Figure 4C**). After IP, the target protein signal was detected through western blotting (**Figure 4D**). At the same time, the silver staining results showed that the difference in protein levels between the IP experimental group and the IgG control group was significant, which further confirmed the success of IP enrichment (**Figure 4E**). Therefore, we further used LC−MS/MS to identify and analyze the binding proteins of S100A9. A total of 243 S100A9-specific binding proteins were identified by LC−MS/MS, of which only TAOK3 and ste20-like kinase (SLK) (**Figure 4F**). The mass spectra of the TAOK3 and SLK proteins are shown in **Supplementary Figure S2A, B**. As shown in **Figure 4G**, Co-IP experiment results showed that the S100A9 protein could not pull down the SLK protein, indicating that there may be no or a weak interaction between the S100A9 protein and the SLK protein. However, the S100A9 protein pulled down the TAOK3 protein, further confirming the interaction between S100A9 and the TAOK3 protein (**Figure 4H**). TAOK3, also known as c-Jun N-terminal inhibitory kinase (JIK), participates in the c-Jun N-terminal kinase (JNK) cascade as a negative regulator. JNK, also known as stress-activated protein kinase (SAPK), is one of the subclasses of the MAPK signaling pathway in mammalian cells (24, 25). In combination with the KEGG signaling pathway of RNA-seq above, we selected the MAPK signaling pathway for further study. S100A9 may regulate MAPK signaling pathway through interacting with TAOK3 protein.

### S100A9 remarkedly up-regulated TAOK3-JNK signaling pathway

**Figure 5:**
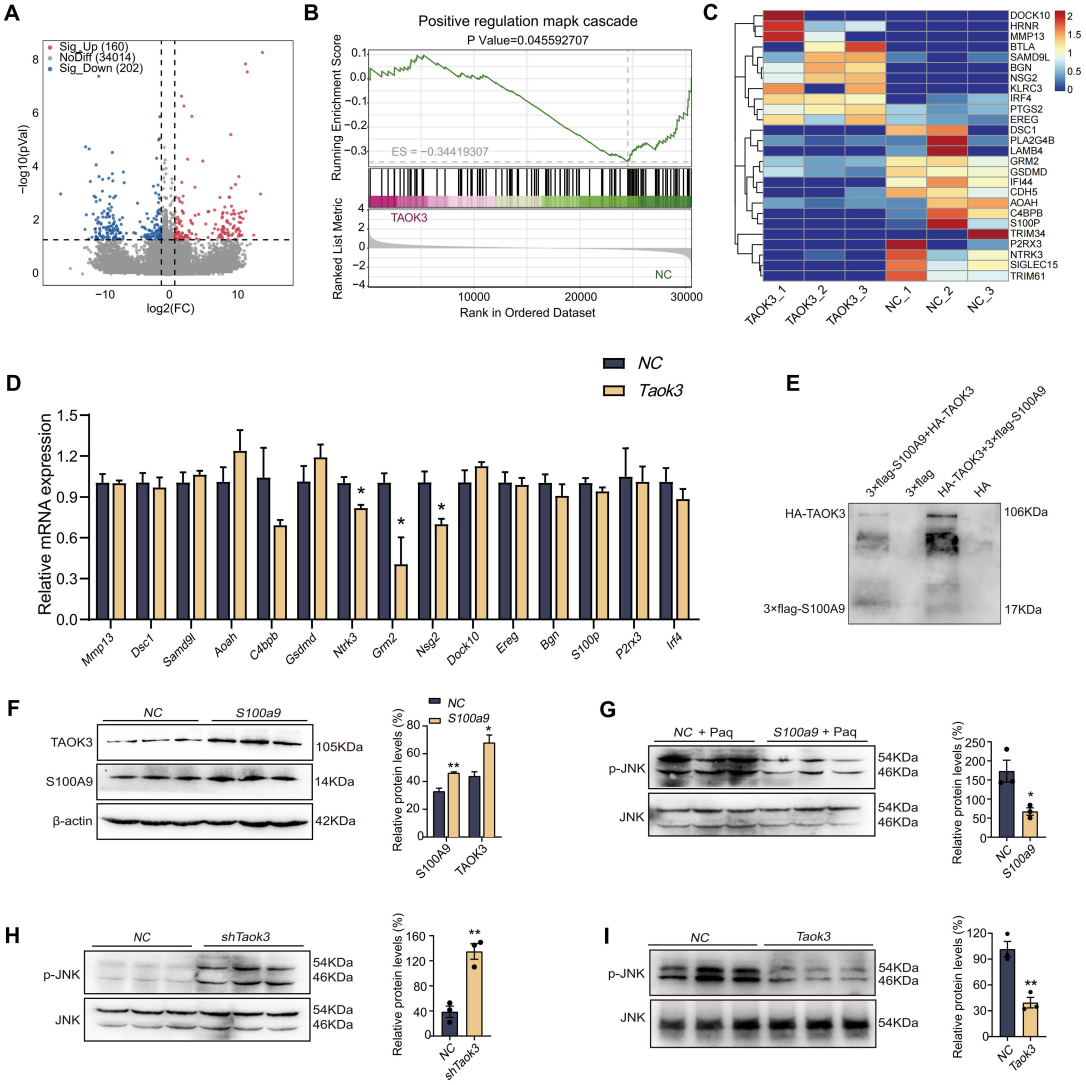
S100A9 remarkedly up-regulated TAOK3-JNK signaling pathway. **(A)** The volcanic map showed the differentially expressed genes of RNA-seq in 293T cells overexpressing *Taok3*. **(B)** The GO analysis results in GSEA analysis showed that *Taok3* negatively regulates the MAPK signaling pathway. **(C)** The heat map showed the inflammation related genes selected from the differential genes of RNA-seq. **(D)** qPCR detected inflammation related genes selected from the differential genes of RNA-seq. **(E)** Pull-down indicated the direct interaction between S100A9 protein and TAOK3 protein. 3×flag peptides are flag-tagged peptides. **(F)** Overexpression of *S100a9* in the RAW264.7 cells upregulated the expression of TAOK3 protein. **(G)** The expression of p-JNK protein was up-regulated in RAW264.7 cells overexpressed with *S100a9* after Paquinimod treatment. **(H)** The expression of p-JNK protein was up-regulated in RAW264.7 cells with low *Taok3* expression. **(I)** Overexpression of *Taok3* down-regulated p-JNK protein expression in 293T cells. Data were presented as the mean ± SEM; *P < 0.05, **P < 0.01 vs. NC group.

To further explore the role of *Taok3* in the MAPK signaling pathway, RNA-seq analysis was performed. As shown in **Figure 5A**, compared with the NC group, 293T cells overexpressing *Taok3* had a total of 362 differentially expressed genes, including 160 upregulated genes and 202 downregulated genes. The results of GO analysis with GSEA showed that *Taok3* negatively regulates the MAPK signaling pathway (**Figure 5B**). As shown in **Figure 5C**, the heatmap shows that genes related to the MAPK pathway and inflammation were screened from 362 differentially expressed genes using GeneCards (https://www.genecards.org). In addition, the genes in the heatmap were validated by qPCR using 293T cells overexpressing *Taok3*. The qPCR results showed that the expression of the *C4bpb*, *Grm2*, *Dock10* and *Ntrk3* genes was in accordance with the transcriptome sequencing results (**Figure 5D**). The above results indicated that *Taok3* negatively regulates the MAPK signaling pathway. As shown in **Figure 5E**, the results from the pull-down experiments showed that the S100A9 protein pulled down the TAOK3 protein. The interaction between S100A9 and TAOK3 protein was thus further confirmed. Western blotting analysis showed that S100A9 significantly upregulated the protein expression of TAOK3 in RAW264.7 cells (**Figure 5F**). Paquinimod is a S100A9-specific inhibitor. Compared with the paquinimod alone treatment group, the overexpression of *S100a9* inhibited the expression of p-JNK protein (**Figure 5G**). Compared with the NC group, the inhibition of *Taok3* in RAW264.7 cells induced an increase in p-JNK protein expression, and p-JNK protein expression might be decreased in 293T cells overexpressing *Taok3*, suggesting that *Taok3* can inhibit p-JNK expression and then negatively regulate the JNK signaling pathway (**Figures 5H, I**). Therefore, S100A9 promote M2 polarization of macrophage maybe through up-regulating TAOK3-JNK signaling pathway.

### Inhibition of S100A9-TAOK3 interaction may be potential treatments for CP

**Figure 6:**
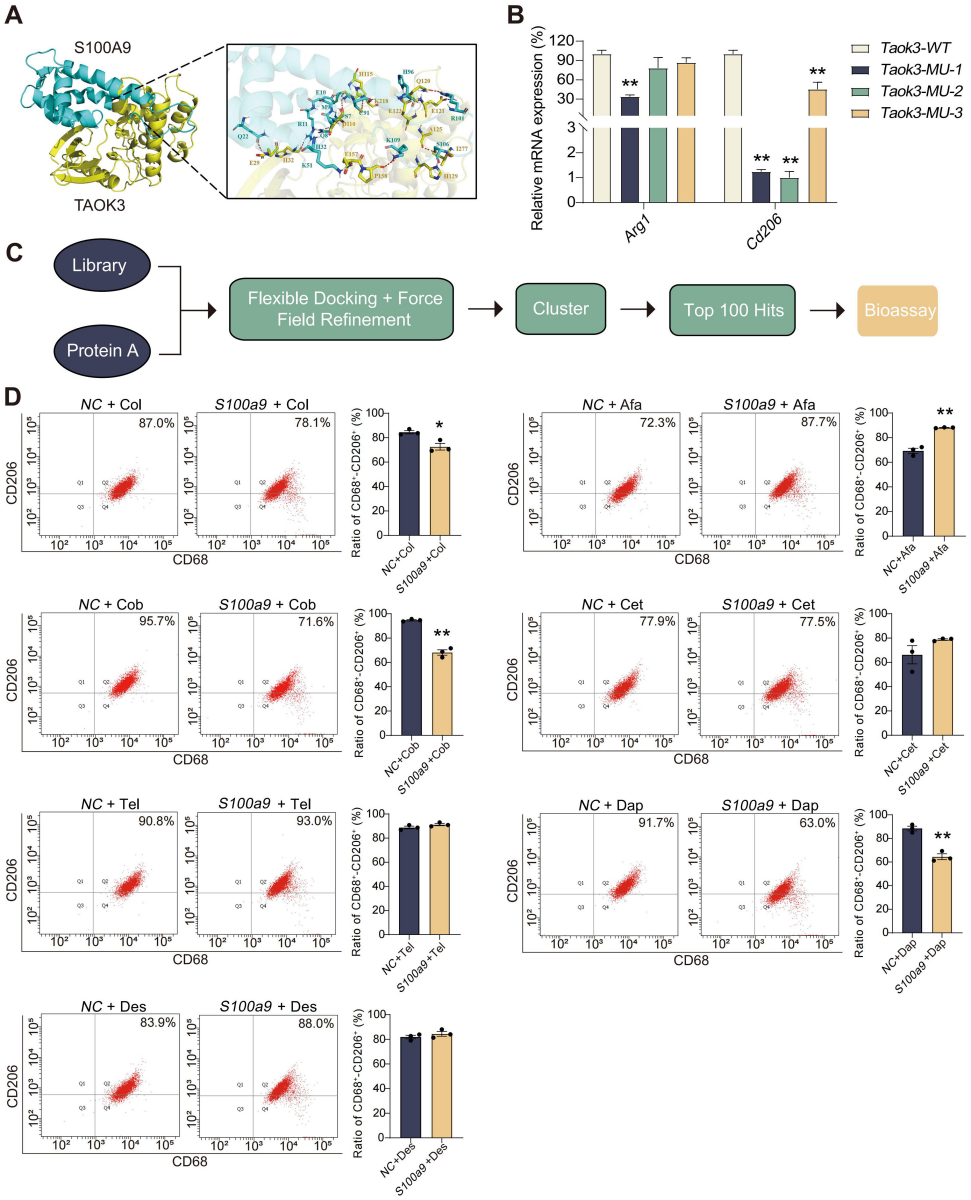
Inhibition of S100A9-TAOK3 interaction may be potential treatments for CP. **(A)** The detail interaction between S100A9 with TAOK3. S100A9 is colored with cyan and TAOK3 with yellow. The red dashes represent hydrogen bond interaction. The blue dashes represent salt bridge. **(B)** qPCR showed that the simultaneous overexpression of S100A9 and three *Taok3*-MU could reduce the mRNA expression of *Arg1* and *Cd206* in the BMDMs, compared with the simultaneous overexpression of S100A9 and *Taok3*-WT, respectively (n=3). **(C)** Flow chart of S100A9-TAOK3 virtual screening of targeted inhibitors. **(D)** Flow cytometry was used to detect the effect of targeted inhibitors on M2 polarization of RAW264.7 cells *in vitro* (n=3). Col: Colistimethate sodium; Afa: Afamelanotide; Cob: Cobamamide; Cet: Cetrorelix acetate; Tel: telavancin hydrochloride; Dap: Daptomycin; Des: Desmopressin. Data were presented as the mean ± SEM; *P < 0.05, **P < 0.01 vs. NC group or *Taok3*-WT group.

To investigate the binding mode of S100A9 with TAOK3, docking simulation studies were carried out. The docking score from ClusPro was -927.9. The interaction between S100A9 and TAOK3 is shown in **Figure 6A** and **Supplementary Figure S3**. The contact list between S100A9 and TAOK3 is shown in **Supplementary Table S4**. Docking simulation studies indicate that the Glu10, Gln8, Gln21, Arg11, Met9, Ser7, Lys51, Cys91, His96, Arg101, Ser106 and Lys109 residues in S100A9 are involved in binding with Lys218, Asp110, Glu29, His32, Glu157, His115, Glu123, Gln120, Glu121, Ala125, Ile277 and Pro158 in TAOK3 through salt bridges and hydrogen bonding interactions. In addition, we constructed three TAOK3-MU plasmids based on the possible binding mode of S100A9 and TAOK3 proteins and found that the *Taok3*-WT plasmid could promote M2-type polarization in the BMDMs compared to the *Taok3*-MU plasmid by qPCR. It is further indicated that the binding of S100A9 protein to TAOK3 protein can promote the M2 polarization of macrophages (**Figure 6B**). Based on the interaction between S100A9 and TAOK3, the top 100 hits for inhibitors of this interaction were finally selected via the virtual screening method (**Figure 6C**), and their structures and docking scores are shown in **Supplementary Figure S4**. To evaluate the efficacy of S100A9-TAOK3-targeted inhibition *in vitro*, the CCK-8 method was used to analyze the cytotoxicity of the top 8 inhibitor compounds, and the maximum nontoxic doses of those 8 compounds *in vitro* were screened as follows: 10 MM colistimethate sodium, 1 MM afamelanotide, 10 MM cobamamide, 1 MM cetrorelix acetate, 1 MM telavancin hydrochloride, 1 MM daptomycin, 10 MM desmopressin. Because actinomycin D at the lowest concentration of 0.001 MM still had cytotoxicity, it was excluded from follow-up studies (**Supplementary Figure S5**). According to the maximum concentration of 7 compounds *in vitro*, the best S100A9-TAOK3 targeted inhibitors of CD206 expression were selected, namely, cobamamide and daptomycin (**Figure 6D**).

### Cobamamide and daptomycin reduce CP by decreasing M2 polarization of macrophages

**Figure 7:**
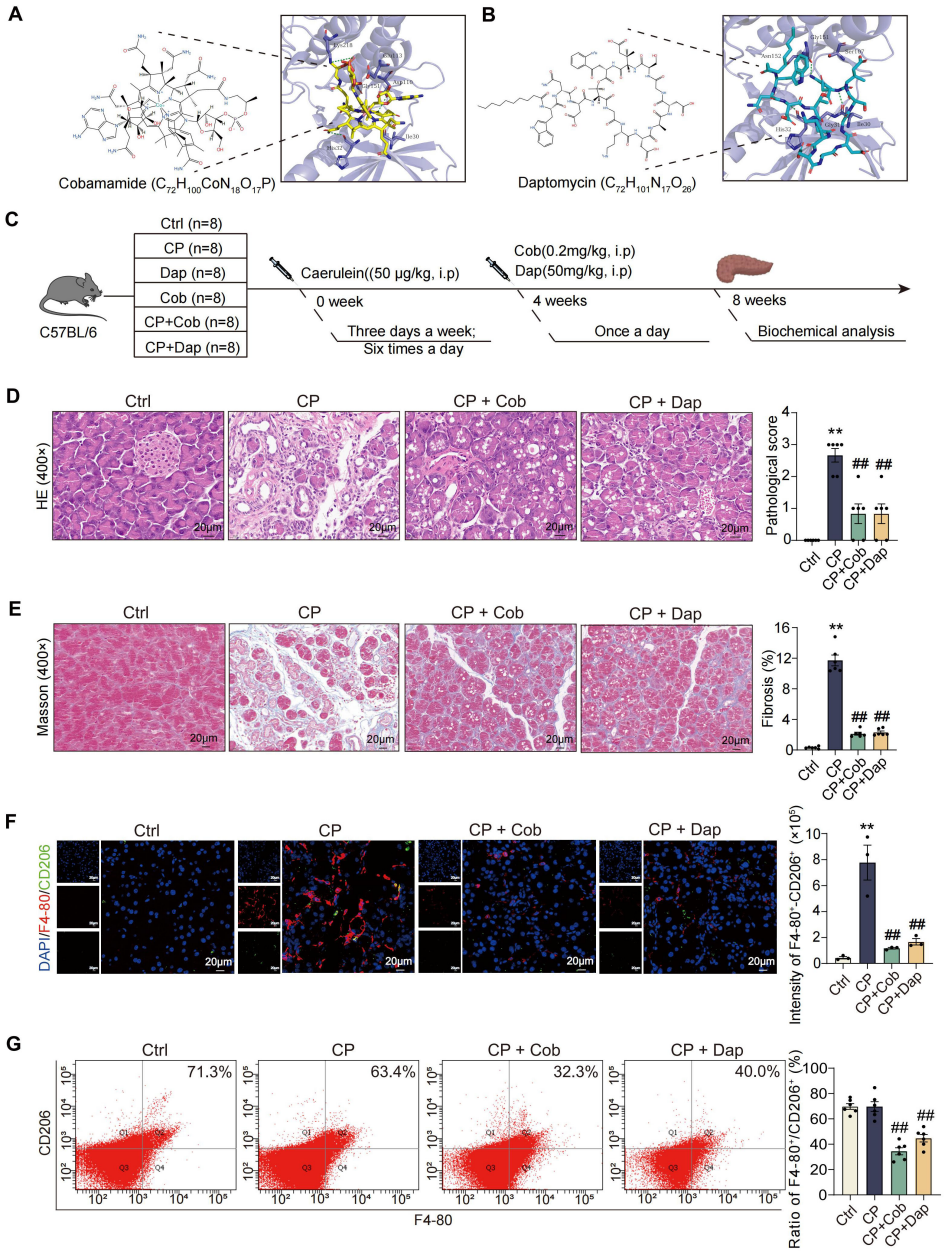
Cobamamide and daptomycin reduced CP by decreasing M2 polarization of macrophages. **(A)** The binding mode of cobamamide with TAOK3 protein. **(B)** The binding mode of daptomycin with TAOK3 protein. **(C)** To verify the toxicology and pharmacodynamics of Cob and Dap in CP in mice. **(D)** HE staining indicates that Cob and Dap can alleviate pancreatic damage during CP of mice (n=6). **(E)** Masson staining indicates that Cob and Dap can alleviate pancreatic fibrosis (blue) during CP of mice (n=6). **(F)** The section of F4-80^+^-CD206^+^ fluorescence double staining showed that Cob and Dap can downregulate the expression of F4-80 protein and CD206 protein in the pancreas of CP mice (n=3). **(G)** Flow cytometry showed that Cob and Dap can reduce the content of CD206 in the spleen of mice during CP (n=6). Cob: Cobamamide; Dap: Daptomycin. Data were presented as the mean ± SEM; **P < 0.01 vs. Ctrl mice; ^##^ *P* < 0.01 vs. CP mice.

The binding mode of cobamamide and daptomycin to the TAOK3 protein is shown in **Figures 7A, B** and **Supplementary Figure S6**, and they both have a suitable spatial complementary relationship with the binding site of TAOK3. To further explore the effect of targeted inhibitors of the S100A9-TAOK3 interaction on CP, cobamamide (0.2 mg/kg/day) and daptomycin (50 mg/kg/day) were used in toxicological and pharmacodynamics experiments *in vivo* (**Figure 7C**). As shown in **Supplementary Figure S7**, compared with levels in the Ctrl group, glutamic pyruvic transaminase (GPT) (hepatotoxicity markers) and urea nitrogen (BUN) and Creatinine (CRE) (nephrotoxicity markers) levels in serum had no obvious changes in both the cobamamide and daptomycin groups, while aspartate aminotransferase (AST) (also hepatotoxicity markers) intensity was increased by daptomycin treatment. In addition, HE staining results of the pancreas, heart, liver, spleen, lung, kidney, brain and intestine also showed that cobamamide and daptomycin had no obvious toxic effect after intraperitoneal injection for 4 weeks (**Supplementary Figure S8**). HE staining was used to evaluate the role of the two targeted inhibitors in CP. As shown in **Figure 7D**, compared with that of Ctrl group, the pancreas of CP mice was severely damaged, and cobamamide and daptomycin treatment significantly reduced the pancreatic damage. Masson staining showed that the area of blue collagen fibrosis in the pancreatic lesions of mice induced by CP was increased but was significantly decreased in the CP + cobamamide and CP + daptomycin groups (**Figure 7E**). The above results showed that cobamamide and daptomycin could reduce the pancreatic injury and fibrosis induced by CP. In addition, F4-80^+^-CD206^+^ double staining results showed that the infiltration of macrophages in the pancreas of CP mice increased (red fluorescence area), and the number of M2 macrophages increased (green fluorescence area) (**Figure 7F**). This result indicated that cobamamide and daptomycin can reduce macrophage infiltration and M2 polarization of macrophages in the pancreatic tissue of CP mice. As shown in **Figure 7G**, the percentage of M2 macrophages in the spleen was detected by flow cytometry, and the proportion was not significant changed compared with that of the Ctrl, but the proportions of M2 macrophages in the spleens of mice in the CP + cobamamide and CP + daptomycin groups were significantly reduced. These results suggested that cobamamide and daptomycin can inhibit the CP-induced M2 polarization of macrophages *in vivo*.

## Discussion

In terms of clinical manifestations and exocrine and pancreatic dysfunction, CP is classified into stages 0 to 4; in the progressive stages of CP (stages 1-2), recurrent AP occurs due to an imbalanced immune response that releases a wide range of inflammatory factors to stimulate pancreatic acinar to ductal metaplasia (ADM) occurrence; during the complications stage of CP (stage 3), persistent destruction of the pancreatic parenchyma by inflammation causes the activation of PSCs and the development of fibrosis (1, 26). Therefore, pancreatic ADM and fibrosis are important pathological changes during the progressive and complication stages of CP, respectively. ADM-producing acinar cells overexpress prostaglandin E2 (PGE2), which synergizes with IL4Rα signaling to slowly transform macrophages in the pancreatic parenchyma from the M1 to the M2 subtype (27). The proliferation and activation of PSCs induced by M2 macrophages promote ECM synthesis, resulting in pancreatic fibrosis through autocrine and paracrine cytokines, such as transforming growth factor-β (TGF-β) and platelet-derived growth factor (PDGF). Activated PSCs in turn secrete inflammatory factors, including IL-4/13, that can promote M1 macrophage transformation into the M2 subtype, leading to more activated PSCs and aggravated pancreatic fibrosis through a positive feedback loop (6). There is a lack of understanding, however, of the mechanisms involved in the crosstalk between the M2-type polarization of macrophages and PSCs.

Our earlier study showed that knockout of the *S100a9* gene in mice significantly reduced the inflammatory response and the damage to the pancreatic parenchyma induced by AP (13). Thus far, CP progression has not been reported to be influenced by S100A9. In the present work, we found that the serum S100A9 level in CP mice was significantly higher than that in Ctrl mice. A significant increase in S100A9 expression was also observed in macrophages infiltrating pancreatic tissue during CP. S100A9, also known as MRP14, is a Ca2^+^ binding protein of the S100 family, it occurs as homodimer, heterodimer, homotetramer, or heterotetramer with S100A8 known as calprotectin (28, 29). In response to inflammation, mature macrophages overexpress S100A9, which forms heterodimers with S100A8 in a divalent ion-dependent manner to enhance macrophage migration into inflammatory lesions by interacting with the cytoskeleton and ECM (30). Our research found that loss of S100A9 significantly decreased the CP-induced macrophage infiltration into pancreatic lesions by inhibiting macrophage adhesion and migration. Binding polarized macrophages can promote their adhesion and migration, and we speculate that S100A9 may have an effect on the polarization of infiltrating macrophages in CP (31).

Previous studies that S100A9 plays a complex role in regulating the polarization of macrophages, which can promote M2-type polarization of macrophages, and also inhibit the M2-type polarization under certain conditions (32–34). S100A9 can promote the polarization of M2 macrophages by binding to receptors on the surface of macrophages to activate specific signaling pathways, such as toll-like receptor 4/nuclear factor-κB (TLR4-NFκB) or phosphoinositide 3-kinase/protein kinase B (PI3K/Akt) signaling pathways (21, 35). In addition, S100A9 can influence the progression and prognosis of liver cancer and glioblastoma by promoting M2-type polarization of macrophages (36, 37). To date, the contribution of S100A9 to macrophage polarization in the progression of CP is unknown. In the current study, we evaluated the effects of *S100a9* on macrophage polarization and the development of CP. First, we found that *S100a9* polarized macrophages exposed to pancreatic inflammatory microenvironments into the M2 subtype to accelerate pancreatic injury and fibrosis during CP, while loss of the *S100a9* gene inhibited macrophage M2 polarization in CP mice and attenuated pancreatic injury and fibrosis. In addition, using an *in vitro* coculture system for macrophages overexpressing *S100a9* and primary PSCs, we confirmed that RAW264.7 cells overexpressing *S100a9* can promote M2-type polarization of macrophages and induce primary PSC activation.

RNA-seq was used to further elucidate the molecular events by which *S100a9* regulates macrophage M2 polarization. KEGG analysis suggested that *S100a9* may regulate the MAPK signaling pathway, which can be divided into three subgroups: JNK, p38 MAPK and extracellular signal-regulated kinase (ERK) (38, 39). To search for downstream molecules of S100A9 that regulate the MAPK signaling pathway, co-IP and LC−MS/MS techniques were used to screen S100A9-interacting proteins. The results showed that the S100A9 protein interacts with the TAOK3 protein, and overexpression of *S100a9* can significantly upregulate the expression of the TAOK3 protein. There are three main protein degradation pathways in eukaryotic cells: lysosomal pathway, autophagy pathway, and ubiquitin-proteasome pathway, which have the ability to replenish the cytosol metabolic library, maintain cellular homeostasis, and affect protein stability and interact with other proteins, respectively (40, 41). At present, the specific mechanism of TAOK3 protein degradation in eukaryotic cells has not been clarified, and there are no reports of the association between S100A9 protein and TAOK3 protein. In this study, we found for the first time that S100A9 protein interacts directly with TAOK3 protein, so we believe that S100A9 may promote the expression of TAOK3 protein through lysosomal pathway, autophagy pathway, or ubiquitin-proteasome pathway. And three protein kinase TAOKs have been identified in mammals, namely, TAOK1, TAOK2 and TAOK3, all of which belong to the serine/threonine protein kinase family (25, 42). It is generally believed that TAOK1 and TAOK2 regulate signaling pathways such as SAPK/JNK, while TAOK1, TAOK2 and TAOK3 regulate signaling pathways such as p38 MAPK and Hippo (25). TAOK3, also known as JNK/SAPK inhibitor kinase, has been reported to be associated with epidermal growth factor receptor kinase substrate 8 (EPS8) in expression library analysis (24). We found that the expression of p-JNK protein was downregulated and upregulated by inhibiting *S100a9* and knocking down *Taok3*, respectively, suggesting that S100A9-regulated macrophage M2 polarization may depend on the regulation of the TAOK3-JNK signaling pathway. At present, the role of TAOK3 in regulating the SAPK/JNK signaling pathway is controversial. *Tassi* et al. showed that TAOK3 inhibited the activity of SAPK/JNK in COS7 cells and reduced its reactive activation to human epidermal growth factor (24). *Kapfhamer* et al. found that the level of p-JNK in the brains of mice increased by inhibiting TAOK3, suggesting that TAOK3 is a negative regulator of the SAPK/JNK cascade (43). In contrast, it has been reported that TAOK3 can activate the SAPK/JNK pathway in osteoblasts (44). The inconsistency of TAOK3 in the SAPK/JNK cascade may be caused by differences in the cell environment. Further research is needed to evaluate the role of TAOK3 in the JNK signaling pathway.

The above studies confirmed that inhibition of the S100A9-TAOK3 interaction may become a key therapeutic target for blocking the development of CP. To determine the interaction between S100A9 and the TAOK3 protein, we carried out molecular docking experiments. The results showed that the salt bridges and hydrogen bonding of the Glu10, Gln8, Gln21, Arg11, Met9, Ser7, Lys51, Cys91, His96, Arg101, Ser106 and Lys109 residues in S100A9 interacted with TAOK3. To obtain candidate drugs with high efficiency and low toxicity, we further used virtual screening technology to screen potential targeted inhibitors of the S100A9-TAOK3 interaction. Based on the above binding sites, we screened the top 100 targeting small molecular inhibitors of the S100A9-TAOK3 interaction from the FDA drug compound library and evaluated the cytotoxicity and pharmacodynamics of the top 8 compounds *in vitro*. Finally, 2 small molecular compounds, cobamamide and daptomycin, showed the best inhibitory activity against macrophage M2 polarization after the maximum nontoxic dose *in vitro*. Daptomycin is a calcium-dependent membrane-bound lipopeptide antibiotic with a 13-amino-acid structure that is a cyclic peptide composed of an n-decanoyl fatty acid chain. It was approved by the FDA in 2003 for infections of complex skin and skin structures or bacterial blood associated with right infective endocarditis (45, 46). In addition, daptomycin can penetrate immune cells, including neutrophils and macrophages, to trigger the immune regulatory response (47). However, the immunomodulatory effect of daptomycin is still unknown. As a lipopeptide, it can directly interact with the lipid membrane of immune cells and may interact with pattern recognition receptors on Toll-like receptors in activated antigen presenting cells (47). Cobamamide is a homolog of vitamin B12 and is used as a drug for nourishing peripheral nerves or treating anemia. Currently, there is little research on the mechanism of cobamamide *in vivo*. To date, there has been no relevant research report on the application of cobamamide and daptomycin against CP. Through toxicological and pharmacodynamic studies *in vivo*, it was found that cobamamide (0.2 mg/kg/day) and daptomycin (50 mg/kg/day) had no obvious hepatotoxicity or nephrotoxicity in mice after 4 weeks of continuous administration, and cobamamide and daptomycin could effectively alleviate the pancreatic parenchymal injury and fibrosis induced by CP. Moreover, cobamamide and daptomycin can also reduce the secretion of TAOK3 by inhibiting the S100A9-TAOK3 interaction, thereby inhibiting the CP-induced infiltration of pancreatic macrophages and their conversion to the M2 subtype.

## Conclusion

In summary, S100A9 is an important driver of pancreatic injury and fibrosis in CP progression by promoting macrophage M2 polarization in a TAOK3-JNK pathway-dependent manner. As targeted inhibitors of the S100A9-TAOK3 interaction, cobamamide and daptomycin can effectively reduce macrophage M2 polarization and pancreatic damage *in vitro* and *in vivo* and are expected to be candidate drugs for blocking the progression of CP in the future.

## Data Availability

The RNA sequencing data presented in the study is publicly available in NCBI repository, accession number PRJNA1187892 and PRJNA1187893.
