# Supplementary figures and images for: Targeted inhibitors of S100A9 alleviate chronic pancreatitis by inhibiting M2 macrophage polarization via the TAOK3-JNK signaling pathway
Source: Front Immunol. 2025 Mar 25;16:1526813. doi: 10.3389/fimmu.2025.1526813 (PMC11979270; doi:10.3389/fimmu.2025.1526813)

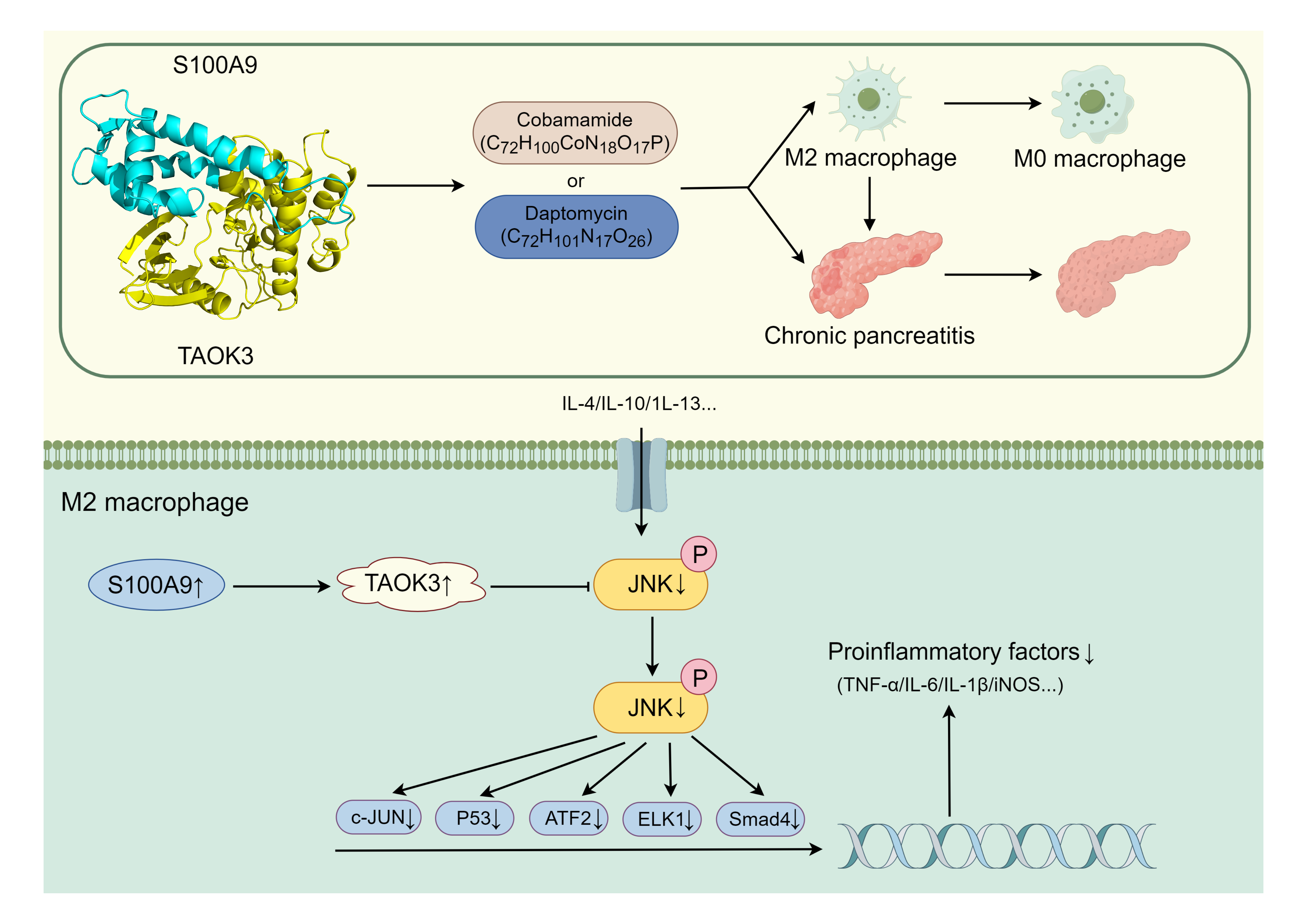

Supplement: Supplementary file 1 [file Image1.png]
