# Supplementary material for: Targeted inhibitors of S100A9 alleviate chronic pancreatitis by inhibiting M2 macrophage polarization via the TAOK3-JNK signaling pathway
Source: Front Immunol. 2025 Mar 25;16:1526813. doi: 10.3389/fimmu.2025.1526813 (PMC11979270; doi:10.3389/fimmu.2025.1526813)
Supplement: Supplementary file 2 [file DataSheet1.docx]

# Supplementary Information

Targeted Inhibitors of S100A9 Alleviate Chronic Pancreatitis by Inhibiting M2 Macrophage Polarization via The TAOK3-JNK Signaling Pathway

Xufeng Tao^1*^, Yu Wu ^1,3*^, Fangyue Guo^2,3*^, Linlin Lv^1^, Xiaohan Zhai^1^, Dong Shang^2,3,4^,

Zhan Yu (🖂)^1#^, Hong Xiang (🖂)^2,3#^, Deshi Dong (🖂)^1#^

^1^ Department of Pharmacy, First Affiliated Hospital of Dalian Medical University, Dalian, 116011, China.

^2^ Laboratory of Integrative Medicine, First Affiliated Hospital of Dalian Medical University, Dalian, 116011, China.

^3^ Institute (College) of Integrative Medicine, Dalian Medical University, Dalian, 116011, China.

^4^ Department of General Surgery, First Affiliated Hospital of Dalian Medical University, Dalian, 116011, China.

^*^These authors contributed equally to this work and share first authorship.

^#^Corresponding author:

Deshi Dong, E-mail: [dongdeshi@dmu.edu.cn;](mailto:dongdeshi@dmu.edu.cn;) Hong Xiang, E-mail: [xianghong0806@163.com;](mailto:xianghong0806@163.com;) Zhan Yu, E-mail: yz700817@163.com

**Table S1** Pathological scoring system used in the present study

| Score | 0 | 1 | 2 | 3 |
| --- | --- | --- | --- | --- |
| Damage | NO | <10% | 10%-50% | >50% |
| ADM | NO | <10% | 10%-50% | >50% |

**Table S2** Primer sequences used for qPCR assay

| Gene | Species | Primers (5’-3’) |
| --- | --- | --- |
| *Cd36* | Mouse | Forward: CTTTGAAAGAACTCTTGTGGGG |
|  |  | Reverse: GTCTGTGCCATTAATCATGTCG |
| *Flnb* | Mouse | Forward: AGGTTGAATGGTGCGAAAGGGAAG |
|  |  | Reverse: TGAAGCGAACGGCATACTTATCTGG |
| *Flnc* | Mouse | Forward: GGACAGAGATCAGCAAGACTC |
|  |  | Reverse: CCTAGAAAATCGCCGAAAACAG |
| *Itga7* | Mouse | Forward: CTGAGAAGAGAAACGTGACTCT |
|  |  | Reverse: ACGATCAAAGCTGTAGAGTGG |
| *Pgf* | Mouse | Forward: CACTTGCTTCTTACAGGTCCTA |
|  |  | Reverse: CACTTCCACTTCTGTTGAGTTG |
| *Itgb5* | Mouse | Forward: TAAGAGAGACTGTGTCGAATGC |
|  |  | Reverse: GTAGCTGAACATCATAACGCAG |
| *Vav3* | Mouse | Forward: GTCAGCCATCTCTAACCTAGAC |
|  |  | Reverse: TAAACTTCCCGTTATTGGCTCT |
| *Bcl2* | Mouse | Forward: GATGACTTCTCTCGTCGCTAC |
|  |  | Reverse: GAACTCAAAGAAGGCCACAATC |
| *Hspg2* | Mouse | Forward: GTTGTCTTCCATGACGTCAAC |
|  |  | Reverse: TAACTGCTGCTCTCGGAATCTT |
| *Igf1* | Mouse | Forward: GAGGGGCTTTTACTTCAACAAG |
|  |  | Reverse: TACATCTCCAGTCTCCTCAGAT |
| *Vwf* | Mouse | Forward: GTGATTTTAACATCTTCGCGGA |
|  |  | Reverse: CAGGAGTTGGCAAAATCATAGG |
| *Actin* | Mouse | Forward: CATCCGTAAAGACCTCTATGCCAAC |
|  |  | Reverse: ATGGAGCCACCGATCCACA |
| *Cd206* | Mouse | Forward: CCTATGAAAATTGGGCTTACGG |
|  |  | Reverse: CTGACAAATCCAGTTGTTGAGG |
| *Arg1* | Mouse | Forward: CATATCTGCCAAAGACATCGTG |
|  |  | Reverse: GACATCAAAGCTCAGGTGAATC |
| *S100a9* | Mouse | Forward: GGAAGCACAGTTGGCAACCTTTATG |
|  |  | Reverse: TGTGTCCAGGTCCTCCATGATGTC |
| *Actin* | Human | Forward: CCTGGGCATGGAGTCCTGTG |
|  |  | Reverse: TCTTCATTGTGCTGGGTGCC |
| *Taok3* | Mouse | Forward: CATAGAGTTGGCGGAGCGGAAG |
|  |  | Reverse: GGAGTCATTCTGAGCGATGTGGTAG |
| *Mmp13* | Human | Forward: GCGGGAATCCTGAAGGAGAATGC |
|  |  | Reverse: TCAAGTTTGCCAGTCACCTCTAAGC |
| *Dsc1* | Human | Forward: GACCTGGAGAAGAAGTAACGGAAGC |
|  |  | Reverse: GGCCACCAACAGTACCAACAGAC |
| *Samd9l* | Human | Forward: TGGCCTGCCTCCTGTTCTGG |
|  |  | Reverse: GTGCTTGCCTGCTTGGACCTG |
| *Aoah* | Human | Forward: CAGCCAACGATGACCAGTCCAG |
|  |  | Reverse: TATTACAGACACCACCAGCACACAC |
| *C4bpb* | Human | Forward: CCTGTGCTGGTGAATGGAGAGTTC |
|  |  | Reverse: CGATTGCTGCCCTTGAGGATGTAG |
| *Gsdmd* | Human | Forward: GCCTCCACAACTTCCTGACAGATG |
|  |  | Reverse: GGTCTCCACCTCTGCCCGTAG |
| *Ntrk3* | Human | Forward: CTGTGTCCTGTTGGTGGTTCTCTTC |
|  |  | Reverse: TGGTGATGCCGTGGTTGATGTG |
| *Grm2* | Human | Forward: ATCACTGGTGTTATTGGCGGTTCC |
|  |  | Reverse: TGGCACTGGTAGAGGCGTAGC |
| *Nsg2* | Human | Forward: ATCGCTGAATTTACGGTCACCATCC |
|  |  | Reverse: AGGCTTTGTAAACCACCAGGAACAC |
| *Dock10* | Human | Forward: TTCCAAGTCAATCAGTCAGCCACAG |
|  |  | Reverse: CAGAATGATCCACAGAGGTCAGCAG |
| *Ereg* | Human | Forward: GTGGGTTATACTGGTGTCCGATGTG |
|  |  | Reverse: ATGTGGAACCGACGACTGTGATAAG |
| *Bgn* | Human | Forward: ACCTCCCTGAGACCCTGAATGAAC |
|  |  | Reverse: AGTAGCGAAGCAGGTCCTCCAG |
| *S100p* | Human | Forward: GCTCAAGGTGCTGATGGAGAAGG |
|  |  | Reverse: AGCCACGAACACTATGAACTCACTG |
| *P2rx3* | Human | Forward: TGTGACATCATCCTGCTCAACTTCC |
|  |  | Reverse: TCAGCGTAGTCTCATTCACCTCCTC |
| *Irf4* | Human | Forward: GCCAAGATTCCAGGTGACTCTATGC |
|  |  | Reverse: GAGGTTCTACGTGAGCTGTGATGAG |

**Table S3** The shRNA sequence of mouse S100A9

| Name | Carrier type | Sequence fragment |
| --- | --- | --- |
| *shNC* | pGPU6/GFP/Neo | **Sense**: CACCGTTCTCCGAACGTGTCACGTTTCAAGA  GAACGTGACACGTTCGGAGAACTTTTTTG |
|  |  | **Antisense**: GATCCAAAAAAGTTCTCCGAACGTGTCAC  GTTCTCTTGAAACGTGACACGTTCGGAGAAC |
| *shS100a9-#1* | pGPU6/GFP/Neo | **Sense**: CACCGCAGCATAACCACCATCATCOGTTCAA  GAGACGATGATGGTGGTTATGCTGCTTTTTTG |
|  |  | **Antisense**: GATCCAAAAAAGCAGCATAACCACCATC  ATCGTCTCTTGAACGATGATGGTGGTTATGCTGC |
| *shS100a9-#2* | pGPU6/GFP/Neo | **Sense**: CACCGCACAGTTGGCAACCTTTATGTTCAAG  AGACATAAAGGTTGCCAACTGTGCTTTTTTG |
|  |  | **Antisense**: GATCCAAAAAAGCACAGTTGGCAACOTT  TATGTCTCTTGAACATAAAGGTTGCCAACTGTGC |
| *shS100a9-#3* | pGPU6/GFP/Neo | **Sense**: CACCGCTGAGCTTTGAGGAGTGTATTTCAA  GAGAATACACTCCTCAAAGCTCAGCTTTTTTG |
|  |  | **Antisense**: GATCCAAAAAAGCTGAGCTTTGAGGAG  TGTATTCTCTTGAAATACACTCCTCAAAGCTCAGC |

**Table S4** The contact list between S100A9 with TAOK3 proteins

| Chain 1 | Residue | Chain 2 | Residue | Interaction type |
| --- | --- | --- | --- | --- |
| TAOK3 | Asp110.OD1 | S100A9 | Gln8.NE2 | Hydrogen bond interaction |
| TAOK3 | Glu29.N | S100A9 | Gln22.OE1 | Hydrogen bond interaction |
| TAOK3 | His32.O | S100A9 | Arg11.NH2 | Hydrogen bond interaction |
| TAOK3 | Lys218.NZ | S100A9 | Met9.SD | Hydrogen bond interaction |
| TAOK3 | Lys218.NZ | S100A9 | Ser7.OG | Hydrogen bond interaction |
| TAOK3 | His115.NE2 | S100A9 | Cys91.SG | Hydrogen bond interaction |
| TAOK3 | Glu123.OE1 | S100A9 | His96.ND1 | Hydrogen bond interaction |
| TAOK3 | Gln120.OE1 | S100A9 | Arg101.NE | Hydrogen bond interaction |
| TAOK3 | Ala125.O | S100A9 | Ser106.OG | Hydrogen bond interaction |
| TAOK3 | Ile277.O | S100A9 | Ser106.N | Hydrogen bond interaction |
| TAOK3 | His129.ND1 | S100A9 | Ser106.OG | Hydrogen bond interaction |
| TAOK3 | Pro158.O | S100A9 | Lys109.NZ | Hydrogen bond interaction |
| TAOK3 | Lys218.NZ | S100A9 | Glu10.OE1 | Salt bridge |
| TAOK3 | Glu157.OE1 | S100A9 | Lys51.NZ | Salt bridge |
| TAOK3 | Glu121.OE1 | S100A9 | Arg101.NH1 | Salt bridge |


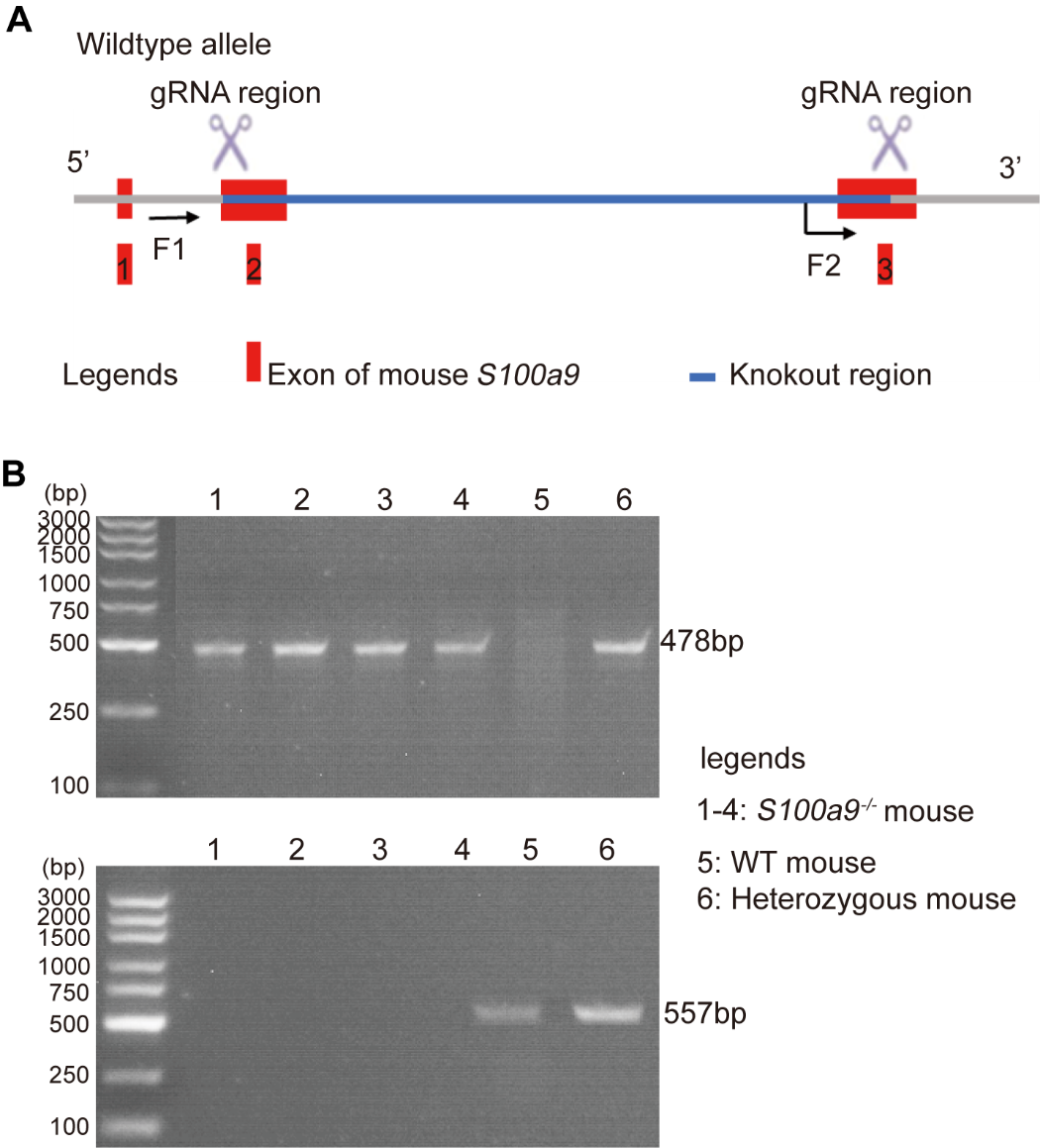


**Figure. S1** Construct *S100a9^-/-^* mice. (A) The schematic diagram of *S100a9^-/-^* mouse was constructed. (B) The genotype of mice was identified by PCR**.**


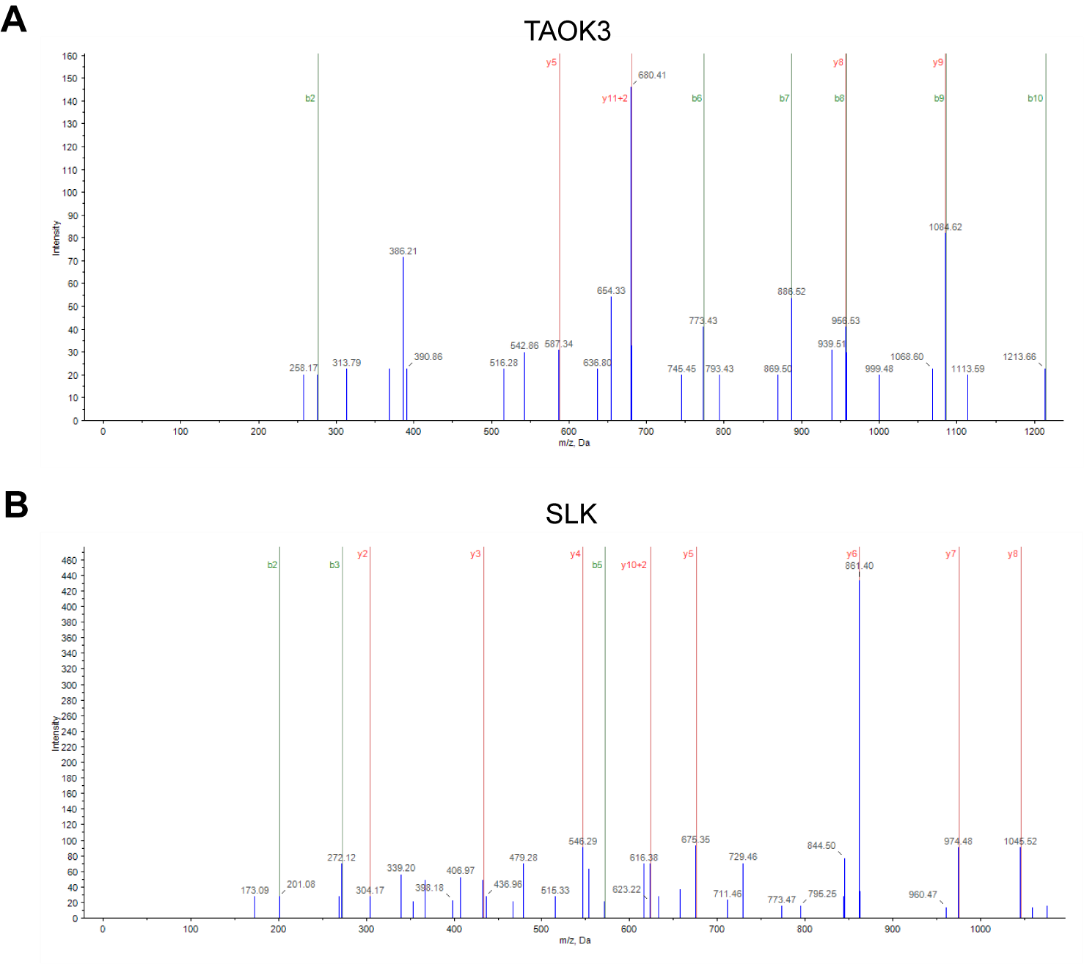


**Figure. S2 The mass spectra of TAOK3 and SLK proteins.** (A) Mass spectrogram of the TAOK3 protein. (B) Mass spectrogram of the SLK protein.


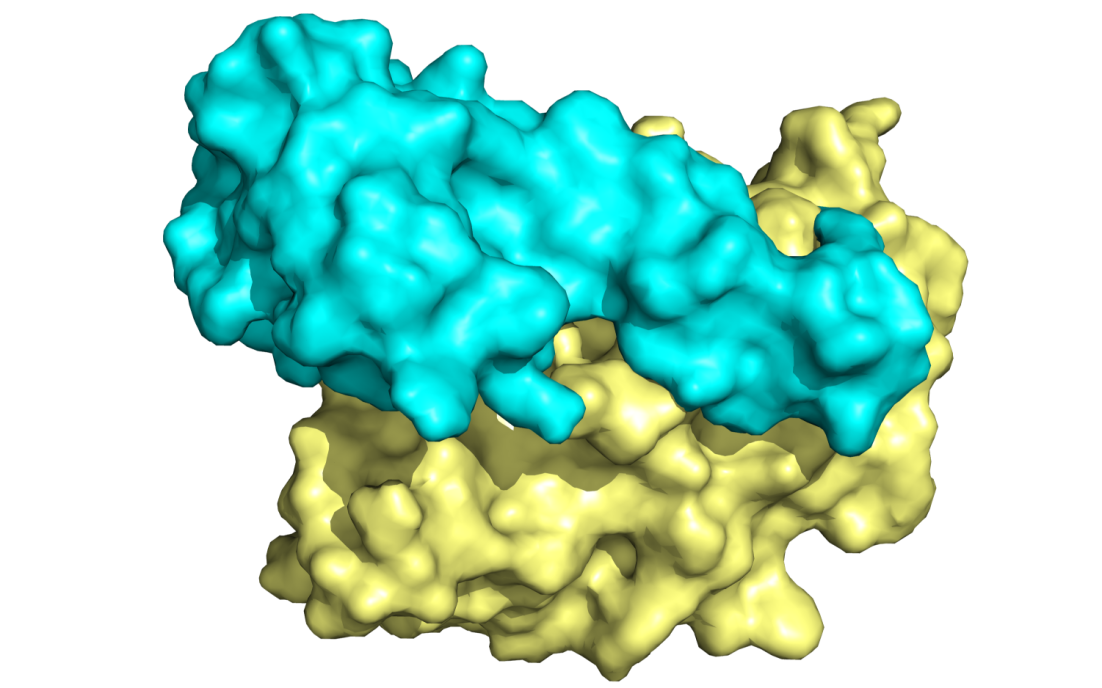


**Figure. S3** Surface bonding model of S100A9 and TAOK3. S100A9 is cyan, and TAOK3 is yellow.


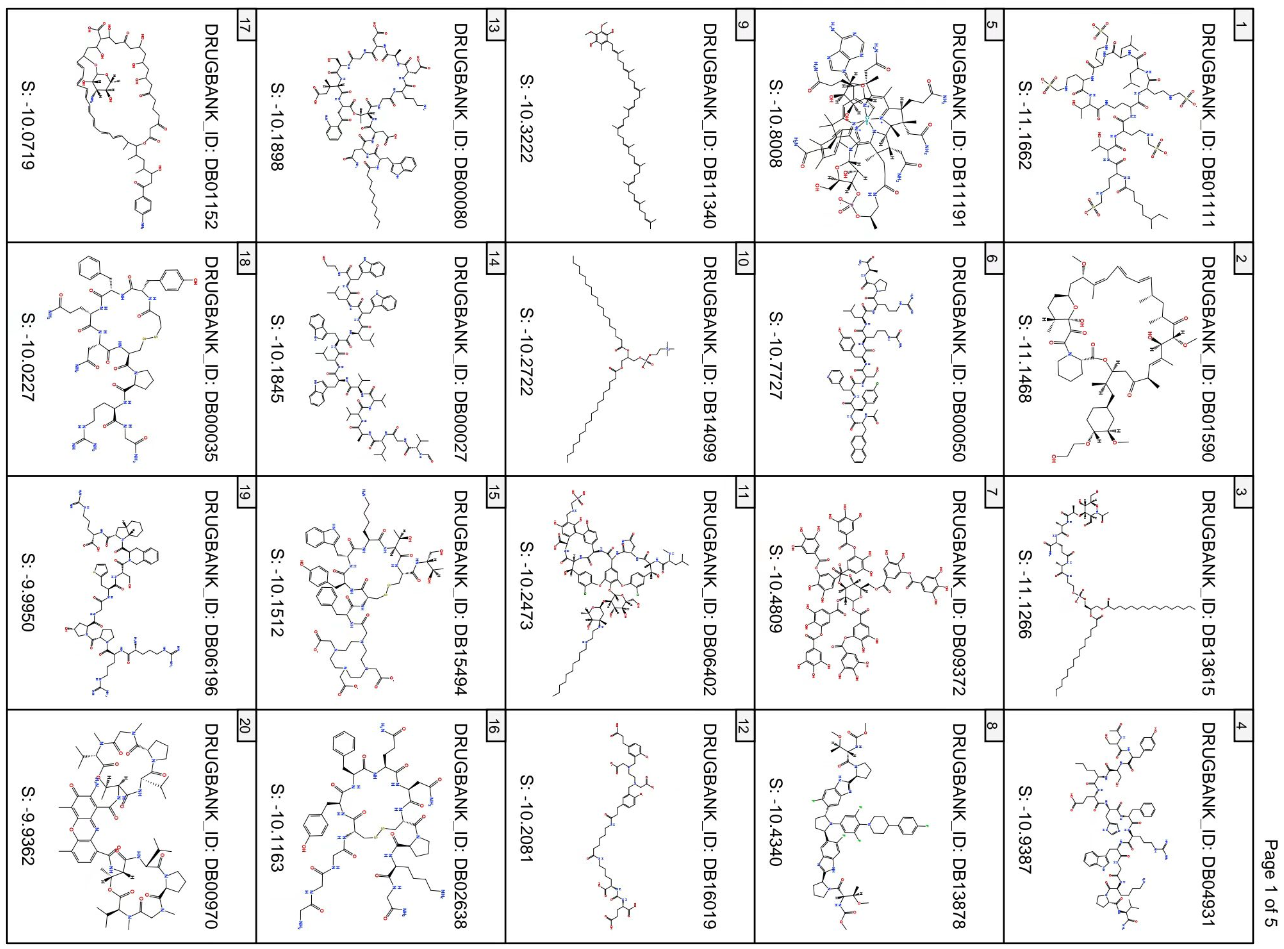

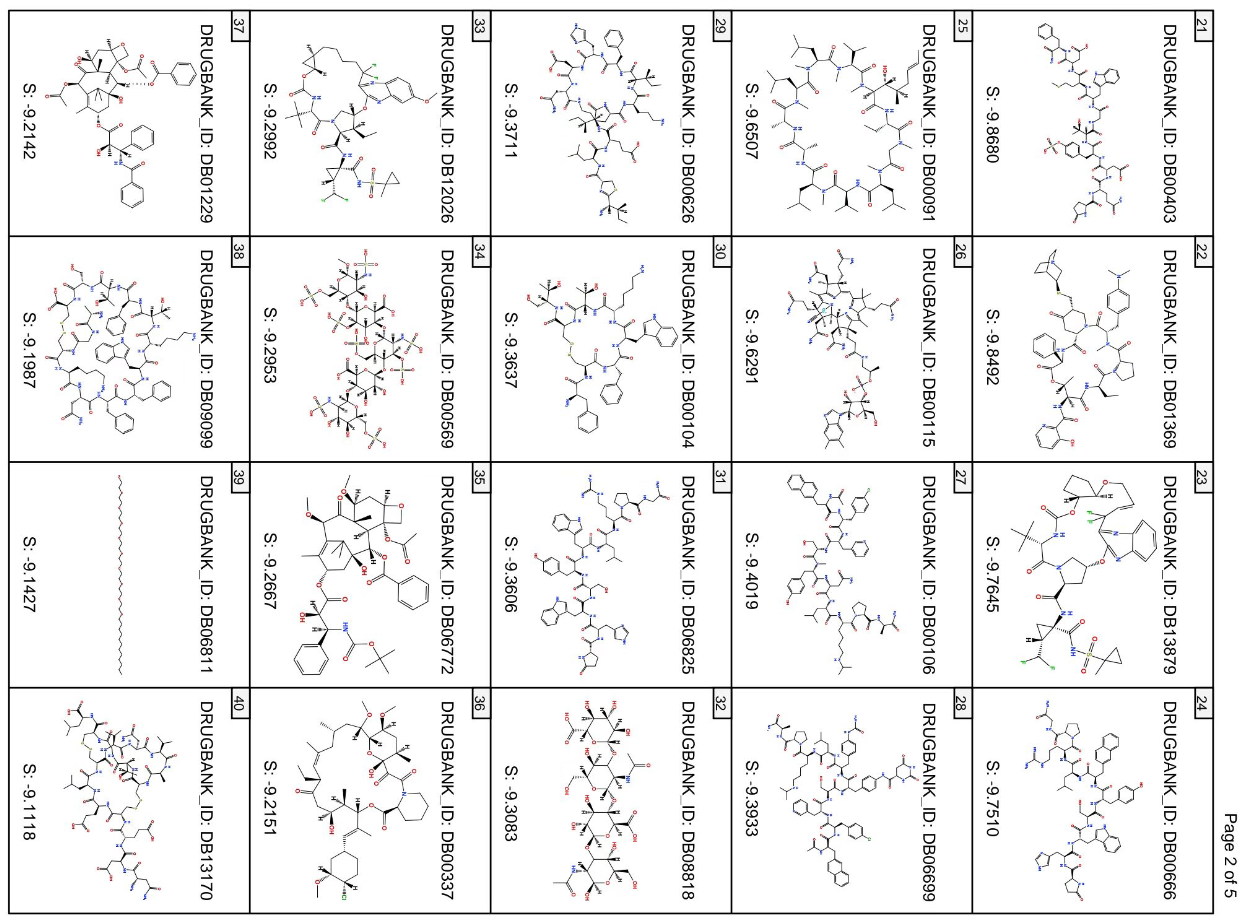

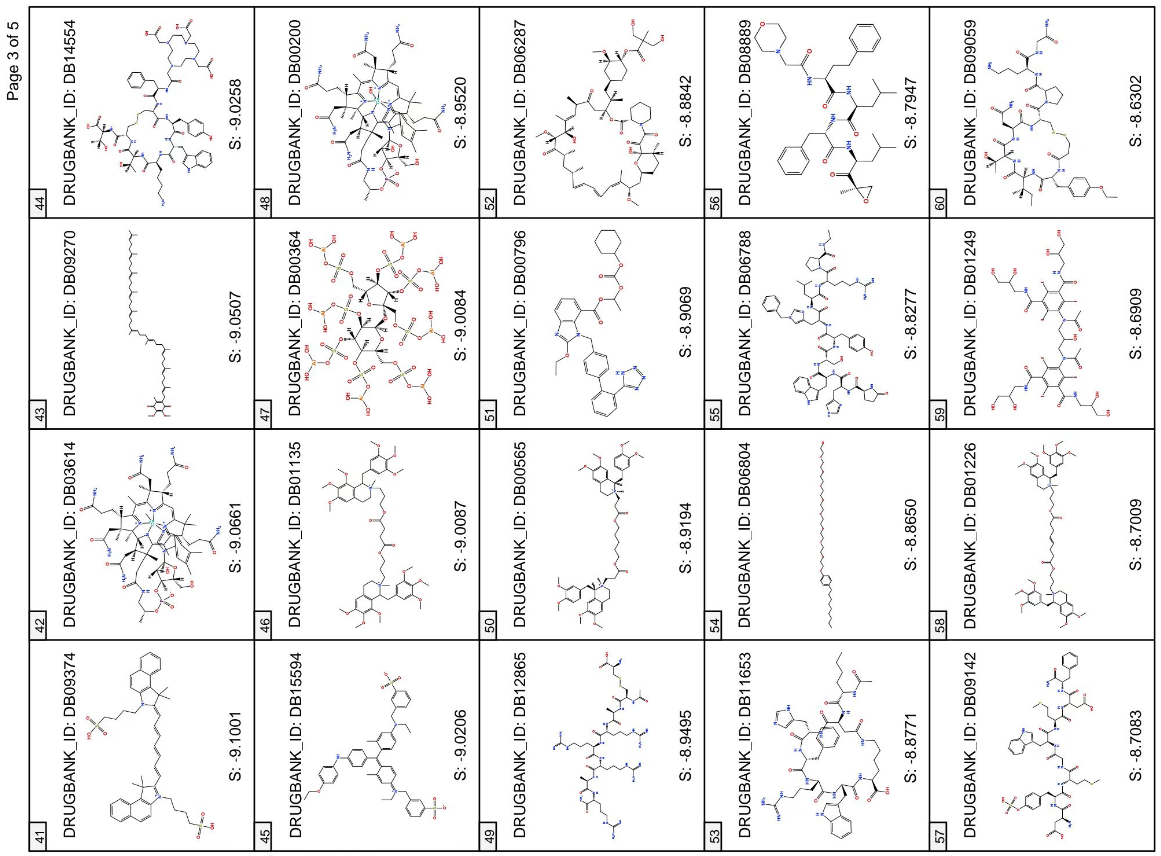

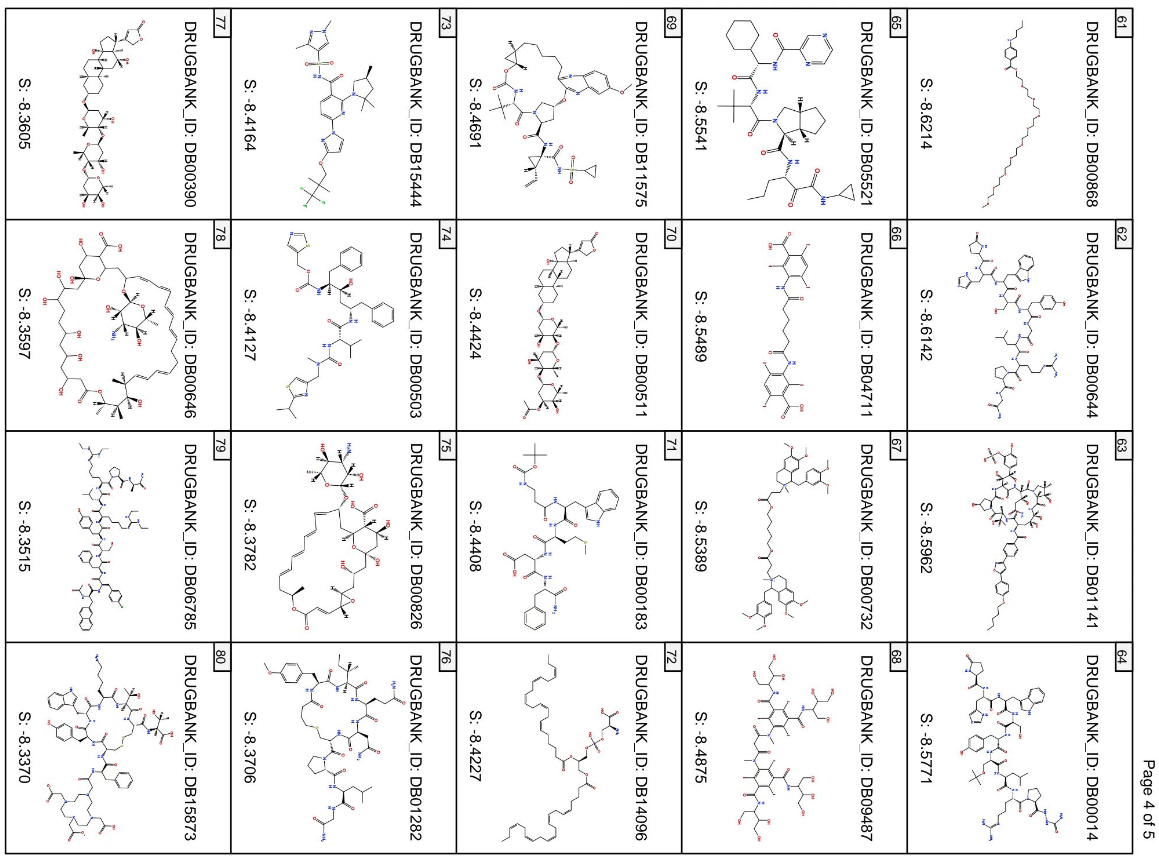

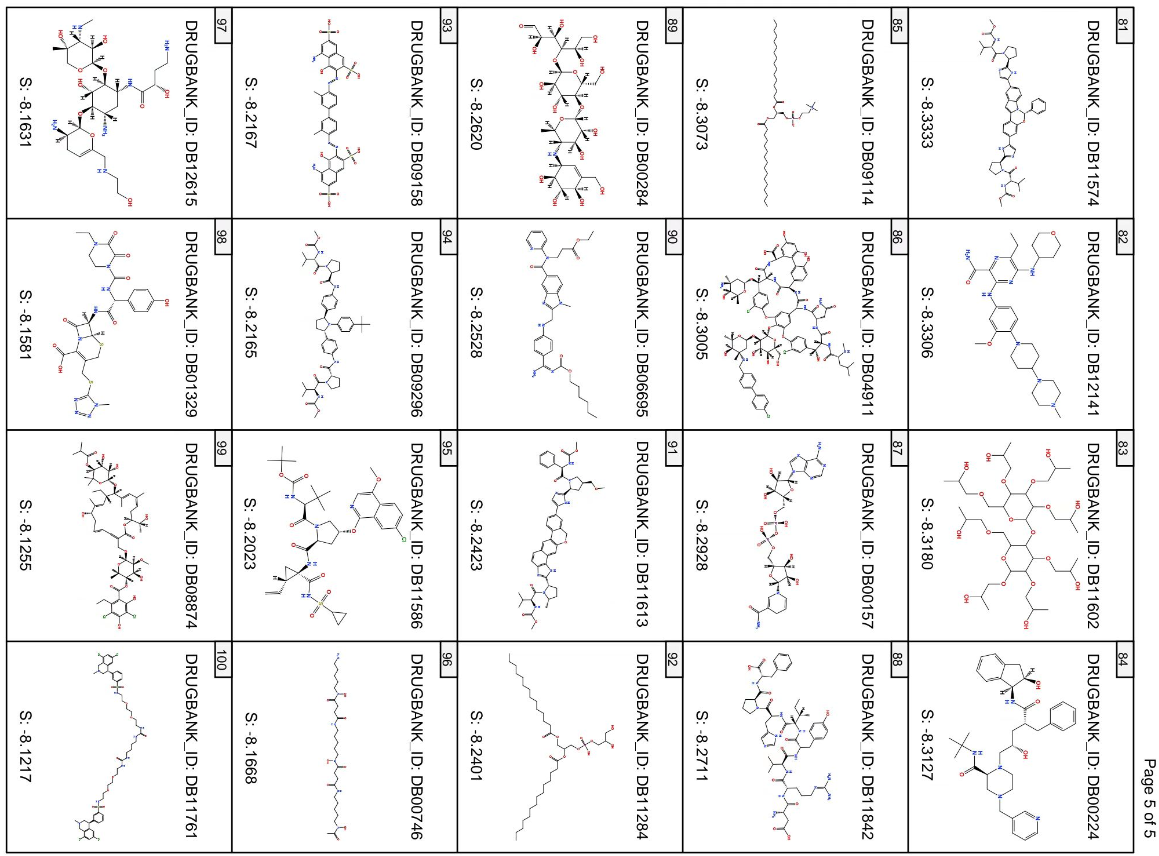


**Figure. S4** Structures and docking scores of the top 100 inhibitors of the S100A9-TAOK3 interaction.

**
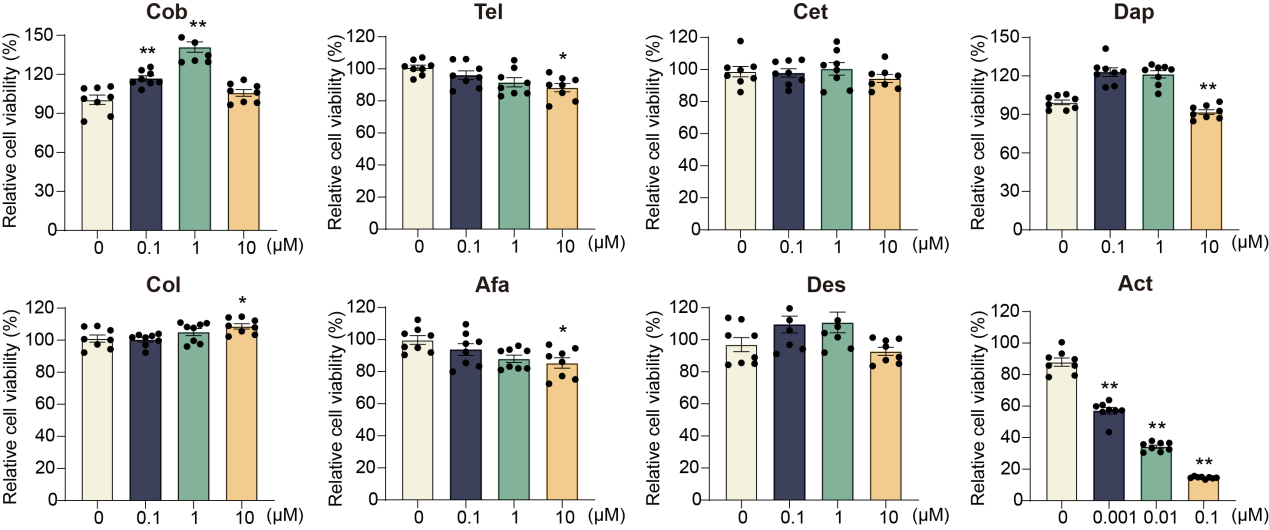
**

**Figure. S5** The CCK-8 method was used to screen the maximum non-toxic concentration of S100A9-TAOK3 targeted inhibitors on RAW264.7 cells *in vitro*. (n=8). Col: Colistimethate sodium; Afa: Afamelanotide; Cob: Cobamamide; Cet: Cetrorelix acetate; Tel: telavancin hydrochloride; Dap: Daptomycin; Des: Desmopressin; Act: Actiomycin D. Data were presented as the mean ± SEM; **P* < 0.05, ***P* < 0.01 vs. NC group.


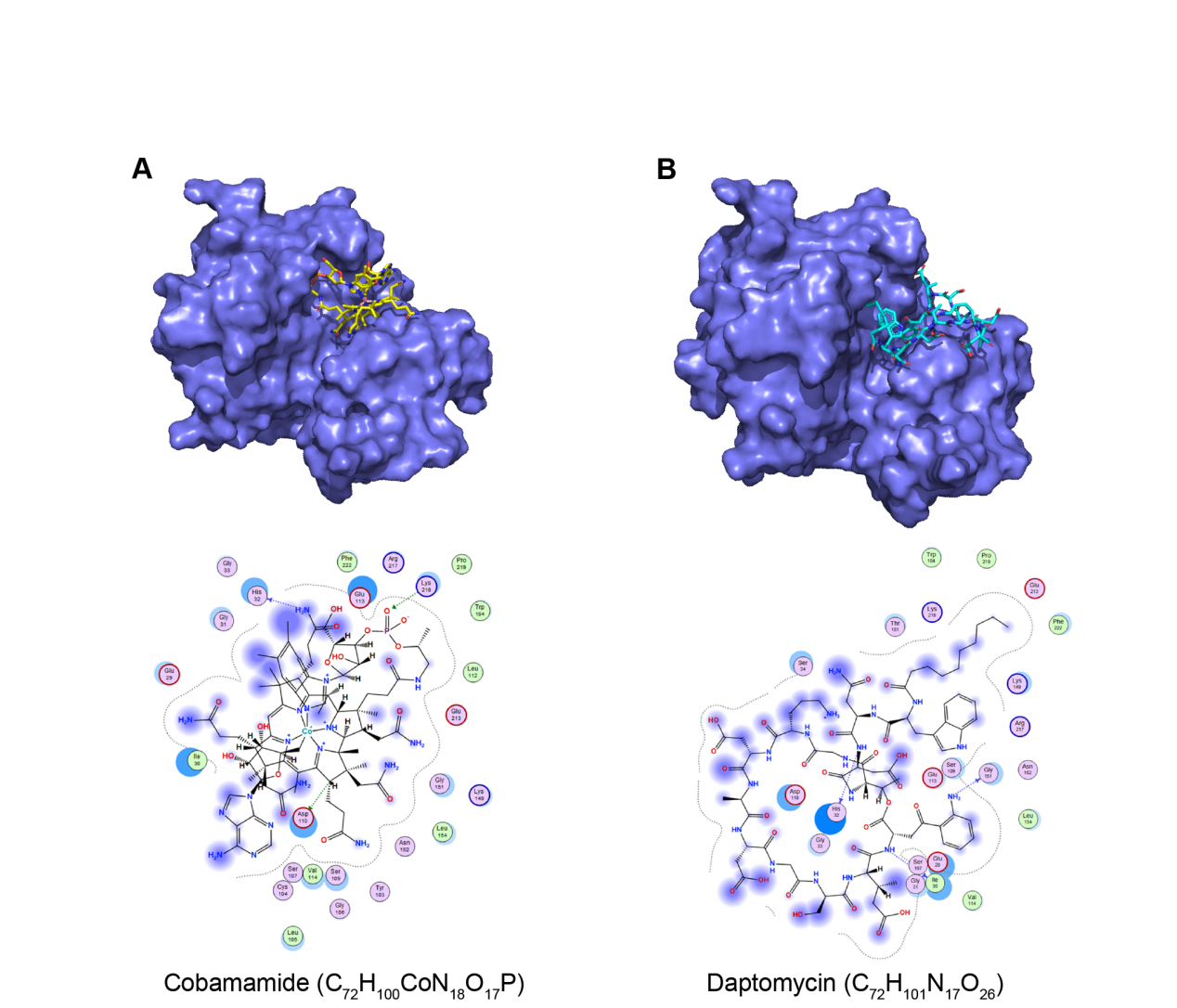


**Figure. S6** Binding models of cobamamide (C_72_H_100_CoN_18_O_17_P) and daptomycin (C_72_H_101_N_17_O_26_) with TAOK3 protein.


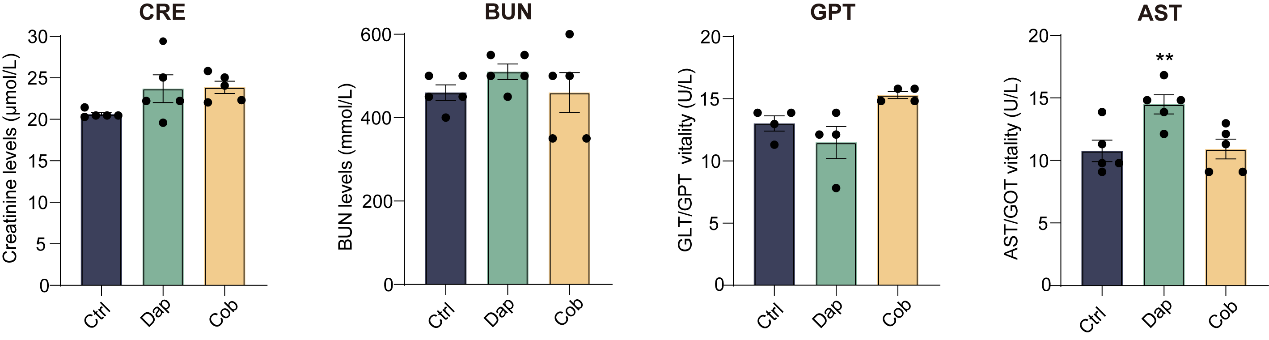


**Figure. S7** The nephrotoxicity and hepatotoxicity of Cob group and Dap group were evaluated by detecting the content of CRE, BUN, GPT and AST in serum of mice (n=4 or 5). Cob: Cobamamide; Dap: Daptomycin. Data were presented as the mean ± SEM; ***P* < 0.01 vs. Ctrl.


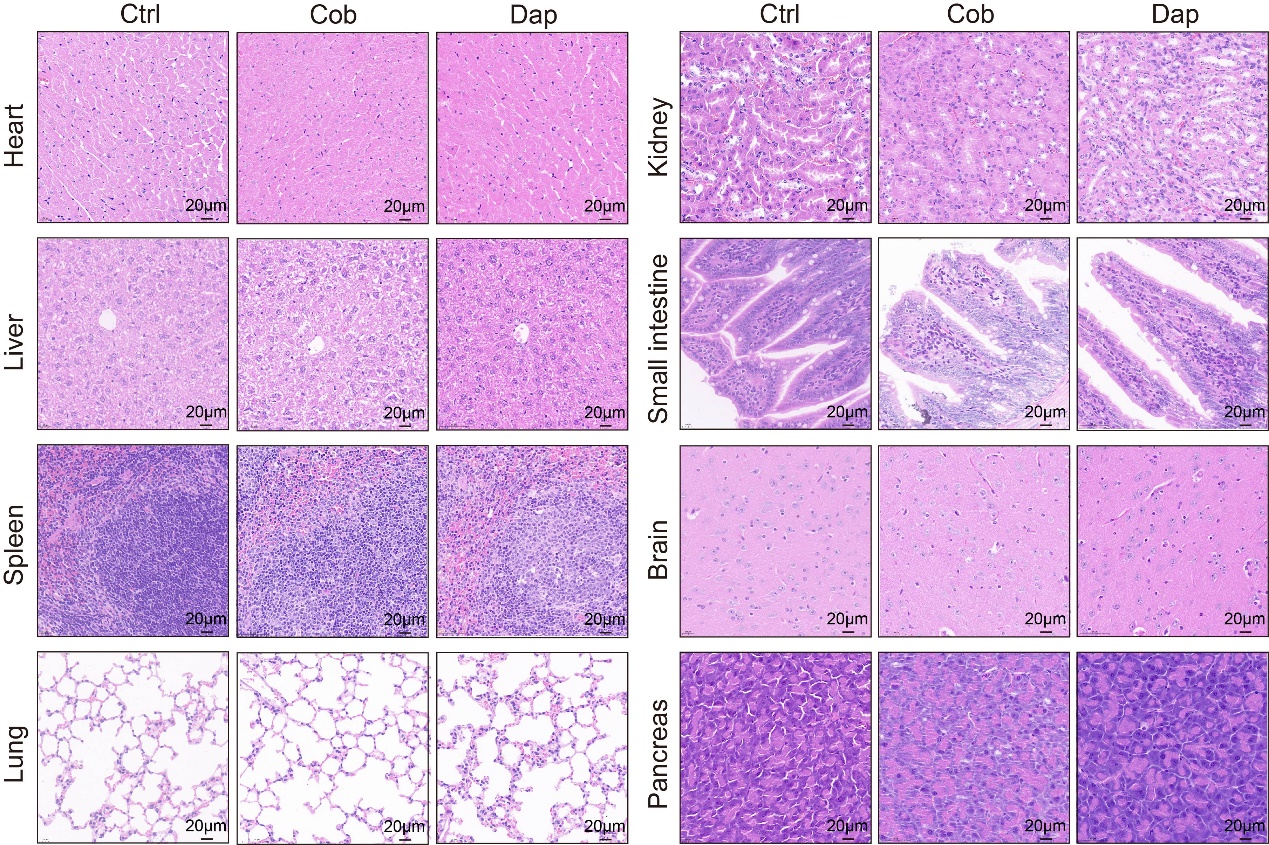


**Figure. S8** HE staining was used to observe the histopathology of heart, liver, spleen, lung, kidney, small intestine, brain and pancreas. Cob: Cobamamide; Dap: Daptomycin.
