# Supplementary material for: Targeted inhibitors of S100A9 alleviate chronic pancreatitis by inhibiting M2 macrophage polarization via the TAOK3-JNK signaling pathway
Source: Front Immunol. 2025 Mar 25;16:1526813. doi: 10.3389/fimmu.2025.1526813 (PMC11979270; doi:10.3389/fimmu.2025.1526813)
Supplement: Supplementary file 3 [file DataSheet2.doc]

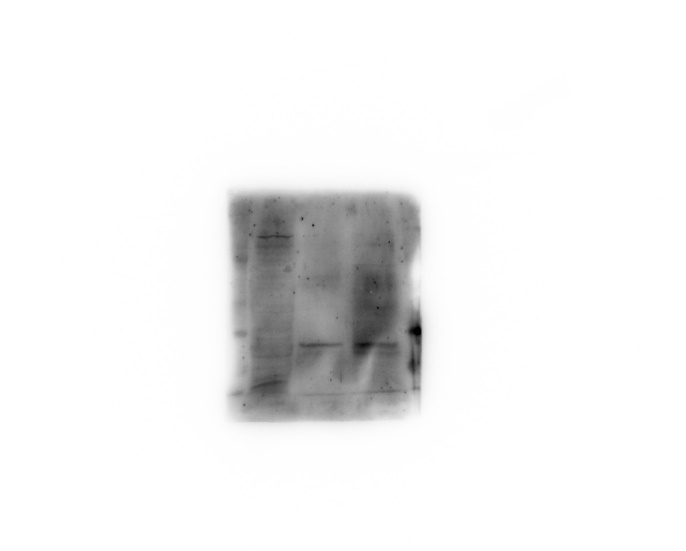


SLK

135KDa

17KDa

Input

IgG

3×flag-IP

3×flag-S100A9

Figure 4G


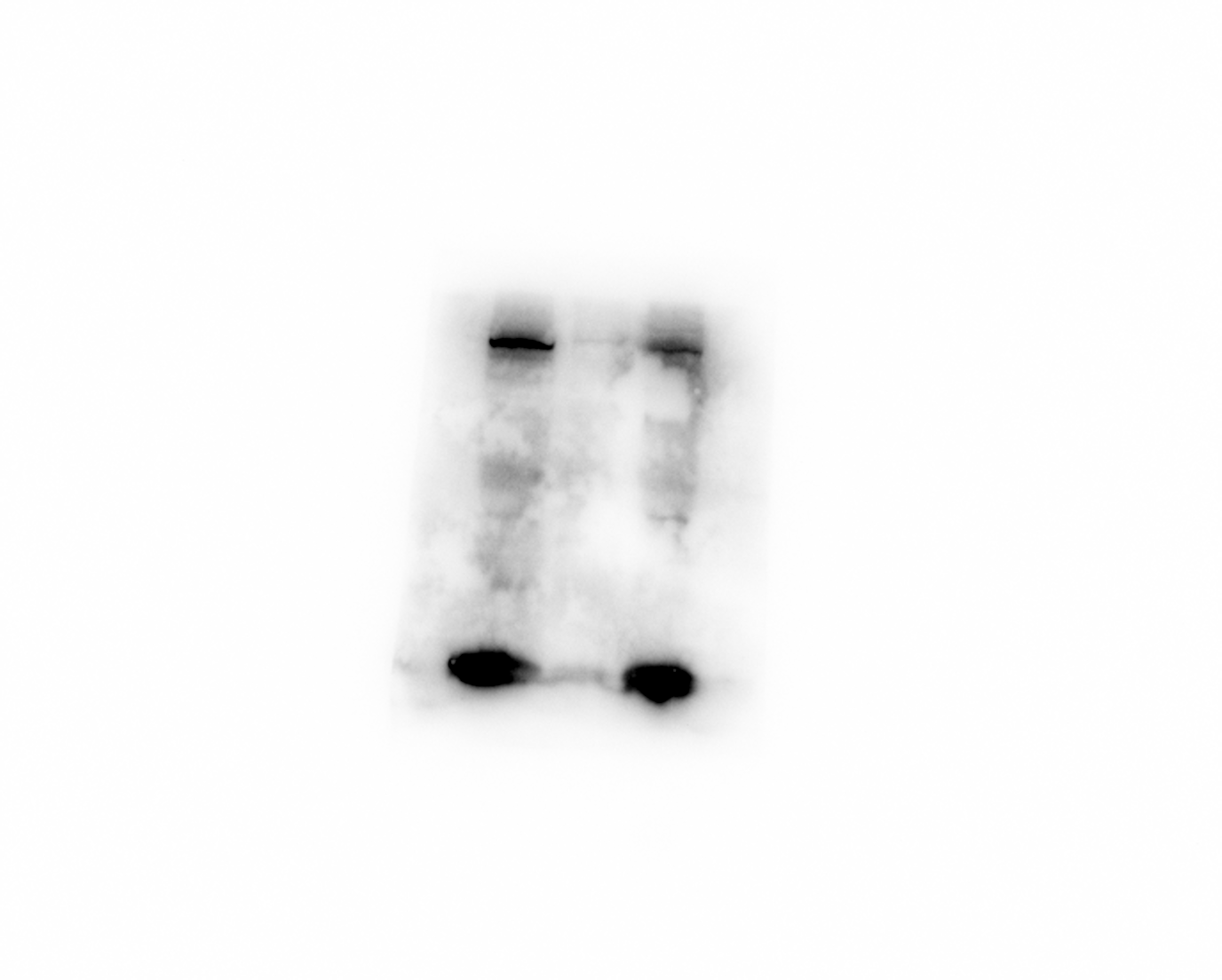


3×flag-IP

Input

IgG

105KDa

17KDa

3×flag-S100A9

HA-TAOK3


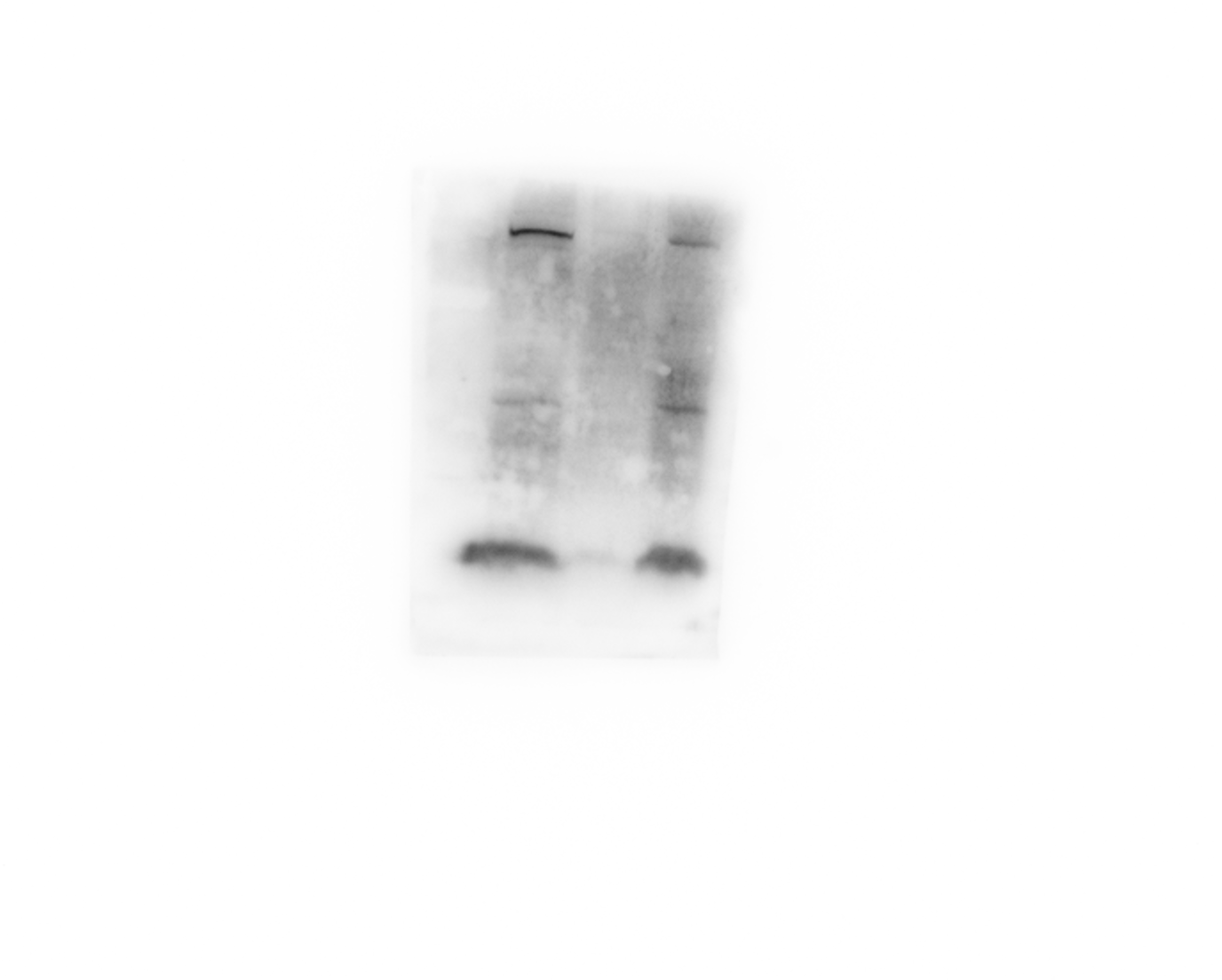


HA-IP

HA-TAOK3

105KDa

Input

IgG

3×flag-S100A9

17KDa

Figure 4H


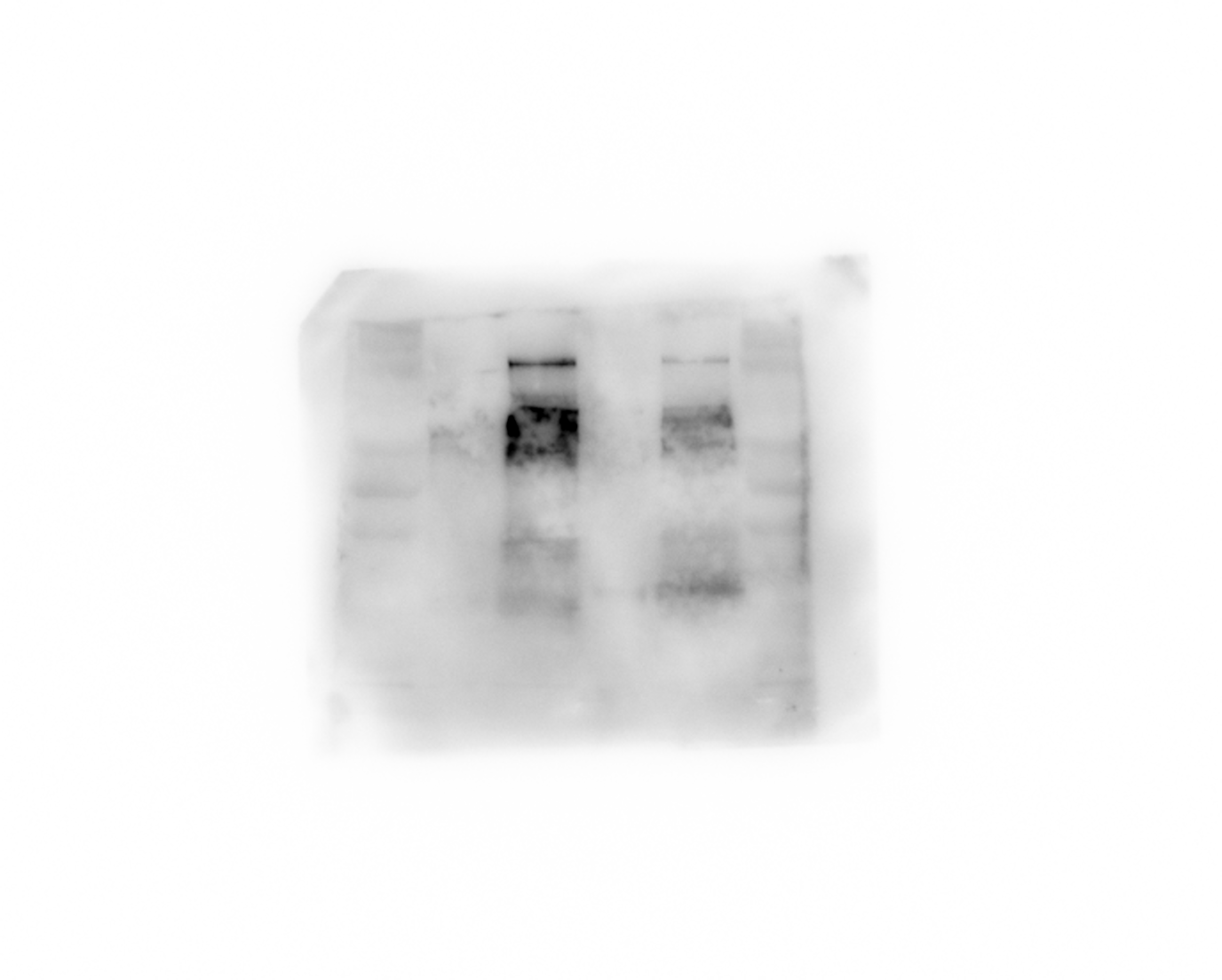


S100A9

105KDa

14KDa

TAOK3

Figure 5E







TAOK3

105KDa

S100a9

S100a9

NC

NC

S100A9

14KDa





42KDa

β-actin

NC

S100a9

Figure 5F







S100a9+Paq

p-JNK

NC+Paq

JNK

54KDa

46KDa

57KDa

46KDa

S100a9+Paq

NC+Paq

Figure 5G







p-JNK

JNK

54KDa

46KDa

57KDa

46KDa

sh-Taok3

NC

sh-Taok3

NC

Figure 5H


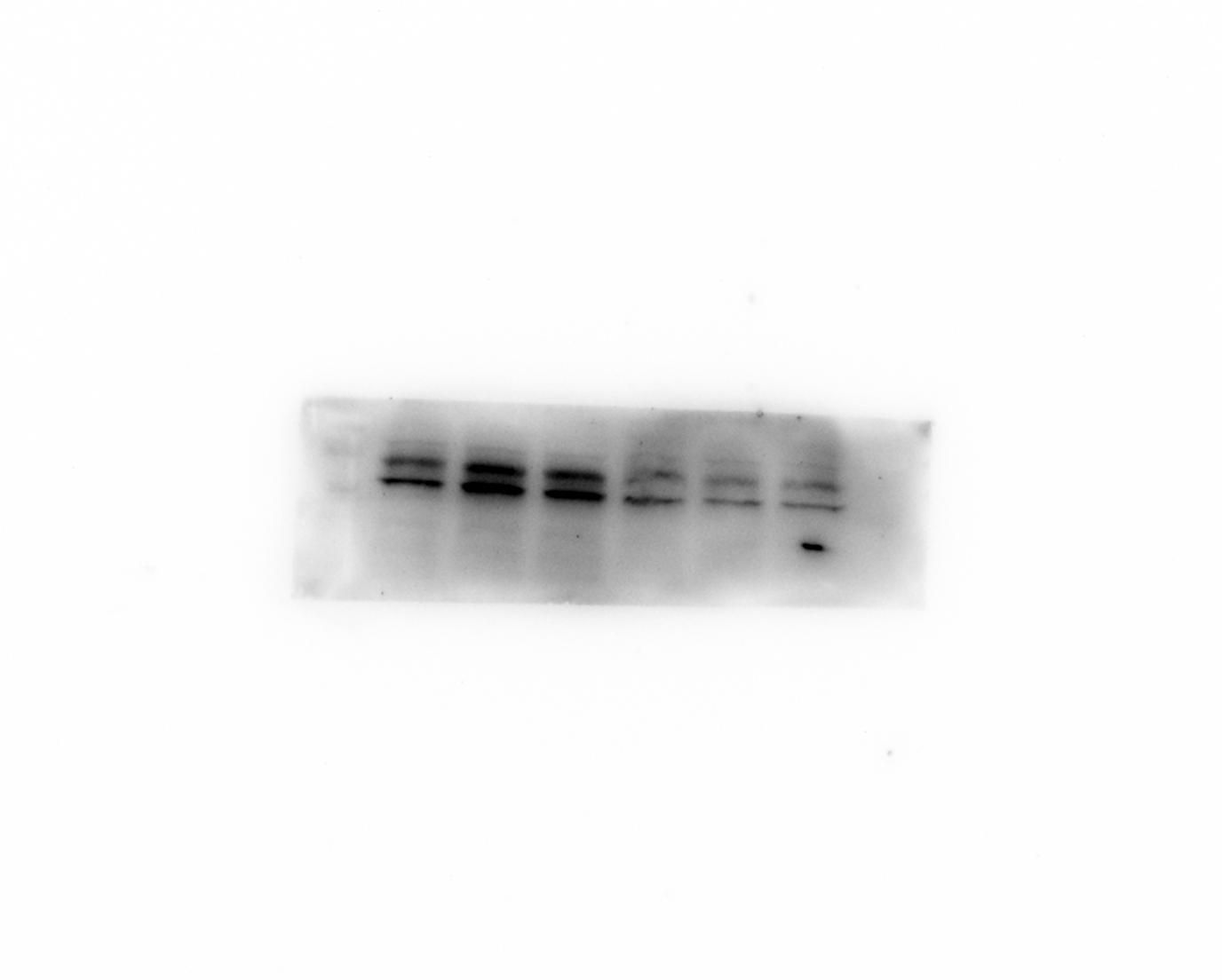

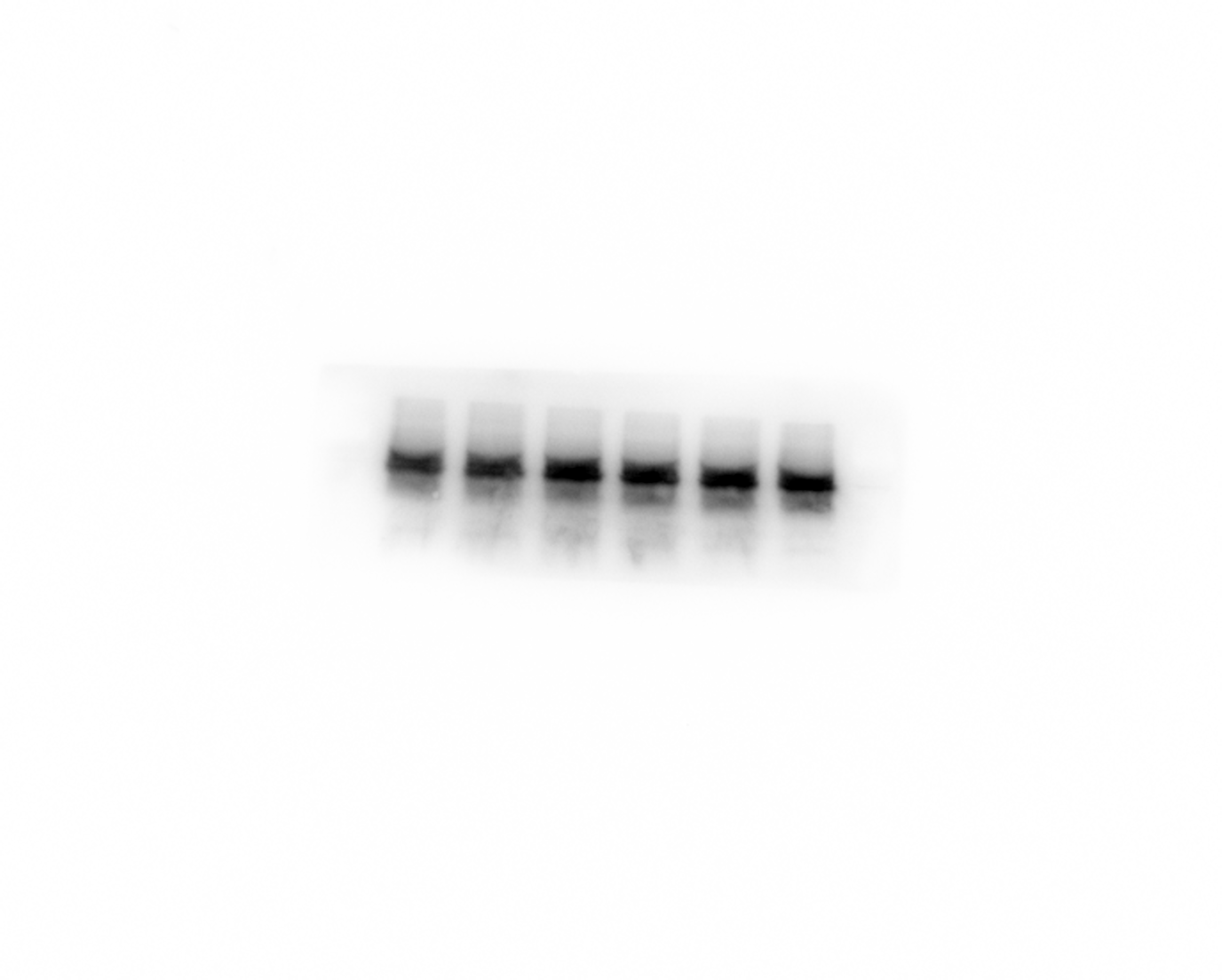


JNK

46KDa

46KDa

p-JNK

Taok3

NC

Taok3

NC

54KDa

Figure 5I
